# A Hybrid Autoencoder YOLO Framework with Spatial Regularization for Rapid Small Maritime Object Detection

**DOI:** 10.3390/jimaging12090433

**Published:** 2026-09-10

**Authors:** M Mohidul Hossain Khan, Qiwei Hu, Radhakrishna Prabhu, Haiyong Zheng, Huagui Huang, Zonghua Liu

**Affiliations:** 1 School of Computing, Engineering and Technology, Robert Gordon University, Aberdeen AB10 7QB, UK; m.khan29@rgu.ac.uk (M.M.H.K.);; 2 National Engineering Research Center for Equipment and Technology of Cold Strip Rolling, Yanshan University, Qinhuangdao 066004, China; 3 College of Electronic Engineering, Ocean University of China, Qingdao 266003, China

**Keywords:** marine security, ship detection, deep learning, real-time object detection, IMO number identification, spatial regularization

## Abstract

Maritime object detection is essential for port-based surveillance, ship tracking, and maritime security. However, practical implementation encounters two primary challenges: significant class imbalance (IMO identification numbers represent only 1.2% of labelled objects) and spatial inconsistency (predicted IMO numbers often appear beyond ship boundaries). Standard detectors and conventional class-balancing strategies fail to adequately address these difficulties, resulting in a persistent mismatch between research validation and practical performance. We present a hybrid autoencoder–YOLO framework with variable spatial regularisation. To the best of our understanding, this is the first methodology to simultaneously address class imbalance and spatial inconsistency in maritime IMO detection via reconstruction-guided learning and differentiable spatial regularisation. The model uses a YOLOv8 encoder shared by both a detection head (designed for ships and IMO numbers), and an auxiliary reconstruction decoder (a SkipDecoder with U-Net-style skip connections). A unique spatial limitation loss provides the physical restriction that each IMO number must be within a ship’s bounding box, using only anticipated boxes. Training occurs in two phases: autoencoder pretraining on the marine dataset, followed by phased joint optimisation with a curriculum schedule for the spatial weighting. In a dataset of 297 annotated images (utilising five-fold cross-validation with a 47-image preserved test set), our comprehensive model achieved 50.1% IMO AP50-95, 97.8% IMO precision, and 94.6% IMO F1-score on the test set, beating the baseline YOLOv8s by +7.8 percentage points, +6.5 percentage points, and +5.2 percentage points, respectively. Ablation studies indicate that reconstruction instruction improves IMO AP50-95 by +4.5 percentage points, while spatial regularisation adds +3.3 percentage points. Although ship detection results in a small compromise (ship AP50-95 decreases from 77.0% to 55.6%), this appears to be practically acceptable given the essential role of IMO numbers as unique ship IDs. Significantly, inference speed improves by 20% (8.0 ms per image on an NVIDIA A100 GPU) relative to YOLOv8s (10.0 ms). Comparisons with leading detectors (RetinaNet, Faster R-CNN, DETR, EfficientDet), all initialised with standard COCO-pretrained backbones and fine-tuned on our dataset, reveal that none achieve an IMO AP50-95 exceeding 42.3%, highlighting the task’s challenge and the accuracy of our design. The proposed framework shows potential to address the gap in practical implementation through reconstruction-based feature learning accompanied by a specified geometric baseline. It is precise, accurate, and fast, aligns with specific physics, and shows promise for real-time maritime surveillance applications, though we acknowledge the need for additional thorough verification across many operational environments.

## 1. Introduction

Marine monitoring systems are essential for worldwide security, trade regulation, and environmental sustainability [1]. Precise identification of marine organisations, including ships and their International Maritime Organisation (IMO) identification numbers [2], is essential for port surveillance, ship monitoring, and regulatory compliance [3]. Despite substantial progress in deep learning-based object detection [4], the shift from controlled experimental validation to real-world applications has remained a significant challenge [5]. A ship’s IMO number is a unique and permanent deck identifier required for its entire operational lifetime. As mandated by SOLAS regulation XI-1/3, this number remains permanently assigned to the ship throughout its entire operational life, and is unaffected by changes in name, ownership, or flag [6]. Ground-based long-distance imaging or drone-based imaging enables ship detection and identification from images using their IMO identifiers [7].

**Figure 1:**
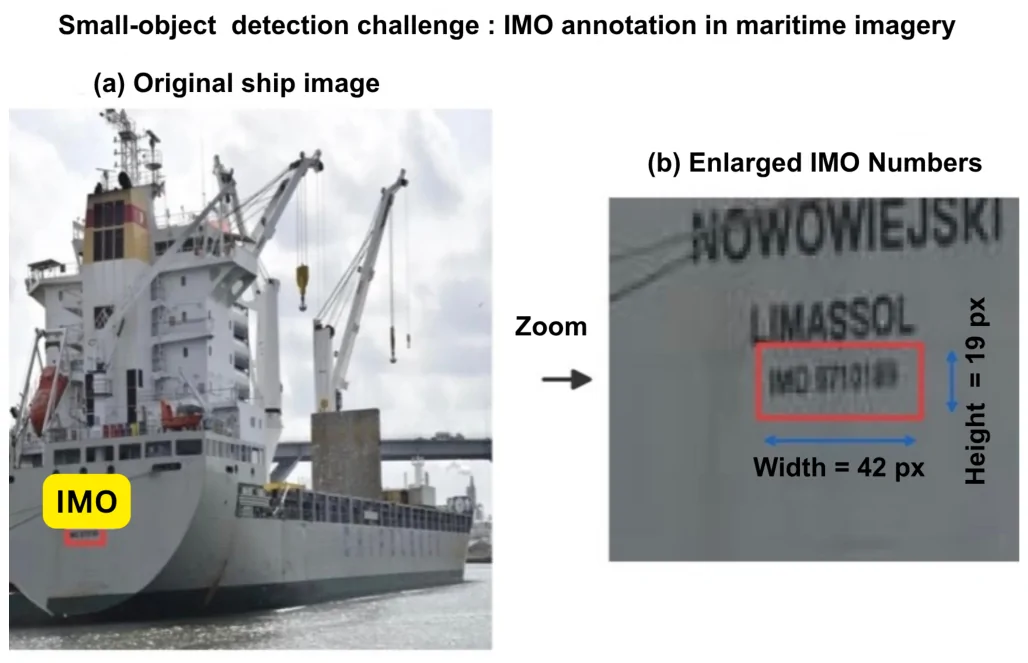
Example of the small-object detection challenge in maritime imagery: (**a**) original ship image with the IMO annotation (red box) and (**b**) enlarged IMO region showing bounding box dimensions of 43 × 19 pixels in a 640 × 640 image.

In recent years, deep learning models such as the YOLO family [8], particularly YOLOv8, have achieved strong performance in detecting and recognising objects in many practical applications [9]. However, applying these models to identify IMO numbers in marine environments presents several difficulties [2]. First, the IMO numbers represent only a small portion of each frame [10]. Across our Folder 5 annotations (640 × 640 pixels), the mean IMO bounding-box height is 17.0 px (median 15.5 px), and 73% of instances are smaller than 20 px in height. Figure 1 shows a representative example in which the IMO box measures only 43 × 19 px, showing the increased difficulty of reliable IMO detection compared to ship detection.

This issue makes identification difficult. A second issue is that IMO numbers vary in format, size, and placement across ships because of differences in ship dimensions, configurations, and operating specifications. Third, due to the shape of the ship’s bow, these digits frequently appear angled or twisted when observed from various angles. Fourth, ocean corrosion and environmental damage blur the numbers over time and reduce their clarity. Finally, a further problem arises from the inherent class imbalance in real-world marine datasets [11]. IMO numbers include only a minor portion of annotated objects relative to ships. Analysis of operational marine surveillance data reveals that IMO numbers account for only 1.2% of marked objects, while ships account for the remaining 98.8%. As a result, standard detection models often cannot detect and identify IMO numbers against the surrounding context [12].

This research presents an organised framework that enhances feature learning under class imbalance and spatial consistency issues when identifying small marine objects. Although the framework performs robustly on our selected dataset, we recognise that additional validation on more extensive and varied datasets is essential before operational implementation. Current methodologies either focus on general object detection without addressing severe class imbalance or implement spatial reasoning regardless of domain-specific limitations, ultimately failing to address both issues simultaneously. Our primary innovation is the integration of: (1) a reconstruction-guided encoder that maintains detailed spatial characteristics for small IMO numbers; and (2) a differentiable spatial regularisation loss that implements the physical requirement that IMO numbers must be located within ship bodies. Together, these components address class imbalance and geographical irregularity, providing a comprehensive solution for maritime IMO detection. The framework includes two primary components:

**(1)**  **Hybrid autoencoder–YOLO framework**: We propose a multi-task architecture which combines YOLOv8 [13] with an additional autoencoder reconstruction branch [14,15]. An inbuilt encoder generates features from the input image. The decoder immediately reconstructs the original image, while an updated detection head executes dual-class detection (SHIP and IMO). This design offers three primary advantages:
  **(a)**  **Enhanced feature representation**: Reconstruction-driven learning captures detailed characteristics that assist in identifying IMO numerical data from the background.
  **(b)**  **Latent regularisation**: The autoencoder decreases overfitting to the majority class (ships) by promoting wide feature learning.
  **(c)**  **Stable training**: Multi-task optimisation enhances convergence, particularly when dealing with highly incorrect data.
**(2)**  **Differentiable Spatial Regularisation**: We provide an innovative loss function that requires an essential maritime constraint, namely, that each identified IMO number must be located within the spatial boundaries of a ship. This limitation serves as an obstacle that reduces predictions of spatial inconsistency while promoting valid ship-IMO connections, building upon previous research on spatial limits and geometric reasoning [16]. This methodology offers three primary benefits:
  **(a)**  **Reduced false positives**: Physically incorrect detections, such as IMO numbers appearing on the water’s surface, are penalised and eliminated.
  **(b)**  **Integration of domain knowledge**: Significant real-world principles regarding ship geometry are immediately incorporated into the training process.
  **(c)**  **Stable convergence**: A curriculum-based weighting technique gradually increases the spatial penalty during training, allowing the model to learn basic detection before applying strict spatial limitations.

## 2. Related Work

### 2.1. Maritime Small Object Detection

Marine object detection has gained greater attention because of its importance in surveillance, navigation, and autonomous systems. Early methods relied on conventional computer vision techniques such as manually engineered features and background subtraction, which worked well only in controlled environments [17]. The growth of deep learning has significantly enhanced detection efficiency in complex environments through convolutional neural network (CNN)-based detectors such as Faster R-CNN, RetinaNet, and SSD as well as transformer-based models such as DETR and Efficient DET [5]. Recent research has utilised these detectors in marine applications, focusing on ship detection in optical and radar data [18].

Beyond broad ship detection, a growing and increasingly complex issue in marine vision is recognising small objects, especially precise targets such as IMO numbers. These items are tiny, often only a few pixels, and are significantly affected by low resolution, motion blur, noise, and insufficient contrast against the ship’s body surface. Although these algorithms perform robustly on benchmark datasets, they often assume balanced data distributions and do not effectively detect small or restricted targets, such as IMO numbers. This places IMO detection within the field of small item detection in complex marine conditions, where maintaining detailed spatial features during feature extraction is essential.

However, most current methodologies address object classification in context and neglect to capture the essential spatial interactions between ships and their specific features. For instance, to minimise error propagation and enhance real-time performance in challenging maritime conditions, USV-based ship identification systems combine detection and recognition into a single pipeline [19]. Similarly, multi-module detection designs such as RT-DETR-based frameworks introduce high-frequency feature enhancement and attention techniques to improve small-object localisation in the presence of boundaries, rotation, and noise [20]. Additionally, sequential detection–recognition frameworks that combine RT-DETR with OCR modules show robustness across a variety of environmental conditions, maintaining excellent accuracy even in fog, water, and low light [21].

Recent methods for identifying small objects in marine environments have shown promising outcomes on publicly accessible benchmarks such as SeaDronesSee and SeaTinySet [22,23,24,25]. These investigations highlight the significance of multi-scale feature representation and improved feature extraction for small maritime targets. However, they focus primarily on object-level maritime targets such as ships, floating objects, and other surface objects rather than small textual areas such as IMO numbers. Additionally, the documented outcomes come from various datasets and tasks, so they cannot be directly compared with the IMO-specific findings of the current investigation. This difference underscores the need for techniques designed for maritime IMO detection, where the significant class imbalance and spatial relationships between IMO areas and their corresponding ship bodies pose additional challenges.

#### 2.1.1. Small Object Detection: Challenges and Approaches

Recognising small objects remains a major challenge in computer vision, especially in marine settings where targets such as IMO numbers consist of only a few pixels. Recent extensive studies have methodically examined the obstacles and approaches in this field. A comprehensive review of deep learning methodologies for small object recognition identified significant obstacles including low resolution, limited feature data, and high sensitivity to interference [25]. The authors classified current methodologies into four viewpoints: enhancing input feature resolution, scale-sensitive training, context-oriented strategies, and super-resolution methods. Similarly, a comprehensive survey on the detection of small and tiny objects emphasised the distinct challenges associated with items smaller than 32 × 32 pixels [26].

In maritime environments, identifying small objects poses further difficulties due to fluctuating sea conditions, inconsistent lighting, and fog or cloud cover. Research on detecting small sea-surface targets has underscored the need for robust feature representations that can accommodate environmental fluctuations [27]. Deep learning methods for detecting small maritime objects have explored strategies such as multi-scale feature integration, attention mechanisms, and super-resolution to improve detection efficiency [28]. However, these techniques primarily focus on typical small items and fail to account for domain-specific spatial constraints, such as the requirement that IMO numbers be located on the ship’s body.

#### 2.1.2. Scene-Text Detection for Maritime IMO Numbers

Given that IMO numbers are fundamentally compact textual objects, the literature on scene-text detection is particularly relevant to this research. Recent progress in scene-based text identification has achieved impressive results on conventional benchmarks via several deep learning frameworks. An extensive overview of scene text comprehension over the last ten years covered topics including advancements in text detection, script recognition, and identification [29]. Numerous impactful techniques have influenced the domain of scene-based text detection. EAST (Efficient and Accurate Scene Text Detector), an uncomplicated yet robust framework that directly forecasts text boxes and adjusts their orientation, has produced swift and precise text detection results in natural environments [30]. CRAFT (Character Region Awareness for Text Recognition) is a system that identifies individual characters and assesses their relationships, allowing for reliable recognition of complex text arrangements [31]. TextSnake is a versatile model for identifying text in various configurations, including horizontal, angled, and curved shapes [32]. Despite these advances, conventional scene-based text detectors assume relatively high resolution and contrast; however, these are rarely present in marine images, where IMO text is often compromised by corrosion, low resolution, and insufficient contrast against the ship’s body. Moreover, these techniques fail to utilise the spatial connections between textual elements and their surrounding frameworks (i.e., ships). Our approach addresses these issues by using reconstruction-guided learning to improve feature representations for impaired text, while spatial regularisation introduces physical constraints relevant to ship-IMO correlations.

### 2.2. Class Imbalance in Object Detection

Class imbalance is a major challenge in object detection, especially when minority classes are largely neglected. Conventional detectors tend to be biased towards majority classes, leading to low recall for rare objects [33]. Various solutions have been suggested to reduce this problem, including data resampling, cost-sensitive learning, and adjusted loss functions. Focal loss was adopted to reduce the influence of easy negatives and enhance learning on challenging cases [34]. Alternative methodologies include class-balanced loss formulations and adaptive reweighting algorithms [35]. Although these strategies improve performance in slightly imbalanced datasets, they often fail under extreme imbalance, where minority classes represent only a small fraction of the data. However, in these cases models may experience class collapse and ignore minority classes during training. This issue is particularly sharp in marine detection, where IMO numbers represent a particularly low-ranking and physically insignificant category. Current imbalance measures are insufficient to ensure reliable detection in real-world scenarios.

### 2.3. Multi-Task Learning and Autoencoder-Based Regularisation

Multi-task learning has been widely studied as a method to enhance feature representation and generalisation through parallel optimisation of related tasks [36]. In object detection, additional tasks such as classification, depth estimation, and reconstruction have been used to improve feature learning and stabilise training [37]. In particular, autoencoders offer an efficient way to create concise and informative representations through image reconstruction [38]. In detection frameworks, reconstruction objectives serve as latent regularisation, promoting the model’s maintenance of precise features essential for identifying small or rare objects. Still, current studies generally apply multi-task learning broadly, and fail to address severe class imbalance or domain-specific limitations such as those in marine settings. Combining autoencoder-based regularisation with current one-stage detectors (e.g., YOLOv8) for imbalanced marine detection remains under investigation.

#### Autoencoder-Enhanced YOLO Frameworks

Recent research indicates that feature learning utilising autoencoders can be combined with YOLO-family detectors for complex image identification challenges. An example is AE-YOLO, a YOLO framework augmented with an attention-guided autoencoder for detecting insulating defects in UAV images [39]. This design integrates lightweight bottleneck autoencoders in the FPN-PAN neck to maintain abnormally sensitive data across multi-scale feature combining. The approach integrates detection losses with a regularisation target based on an autoencoder. These findings show that reconstruction-oriented feature learning can enhance YOLO-based detection by preserving meaningful visual representations in difficult and imbalanced detection contexts.

Previous research has investigated a clear connection between YOLO and autoencoders for various visual applications. An example system uses image extraction based on object detection, wherein YOLOv4 detects relevant data and an autoencoder converts the output object representations into simple hash codes [40]. Tehis approach showed competitive recovery efficiency across three benchmark datasets, indicating the potential of combining YOLO-based object representations with autoencoder-driven feature compression. At the same time, these investigations show the practicality of integrating YOLO-based detection with autoencoder-driven feature learning or reconstruction. However, the aims and application areas of this studies differ from those of the current study, and they do not consider integrating reconstruction-guided feature learning with a domain-specific spatial constraint for maritime IMO identification. Maritime IMO numbers pose a unique difficulty due to their small textual dimensions and their representation as a significantly imbalanced minority class, with their legitimate placement being physically limited by the associated ship bodies. Therefore, the current approach advances YOLO–autoencoder integration by using reconstruction as a feature preservation strategy within the detection architecture and by incorporating differentiable spatial regularisation to ensure ship–IMO spatial consistency.

### 2.4. Spatial Limitations and Geometric Reasoning

Researchers have explored combining spatial restrictions with deep learning models across multiple domains to improve prediction consistency and ensure physical validity [41]. Proposed methods for modelling dependencies between objects include geometric restriction losses, attention mechanisms, and relational reasoning networks. In object detection, many methods use spatial priors or context modelling to improve localisation precision and reduce false positives [42]. However, these techniques mainly emphasise general spatial relationships and fail to specify strict domain-specific constraints.

In marine settings, a primary limitation is that IMO numbers must remain within a ship’s spatial boundaries. Despite its importance, detection models rarely build this limitation directly into their design. As a result, current techniques may produce objectively incorrect predictions, such as identifying markers outside ship boundaries. Three principal limitations in the existing literature may be identified from the previously mentioned review:

1. **Insufficient robustness to substantial class imbalance**: Current tiny object detection techniques prioritise general objects and fail to tackle extreme class imbalance, where IMO numbers represent only 1.2% of annotated items. Focal loss [43] and class-balanced losses [35] improve performance on moderately unbalanced datasets, but fail in extreme imbalance when the minority class accounts for fewer than 2% of annotations.
2. **Restricted utilisation of reconstruction-guided learning**: While autoencoder-driven regularisation has been investigated in multi-task learning [15,37,44], its integration with one-stage detectors for imbalance-sensitive feature enhancement remains largely unexplored, especially in maritime settings where minute text objects require careful feature preservation.
3. **Absence of domain-specific spatial limitations**: Current spatial reasoning methodologies [41,42] emphasise broad associations and fail to specify strict domain-specific limits. No current technique explicitly requires the geometric incorporation of IMO numbers within ship structures, leading to physically mismatched predictions when IMO detections extend beyond ship limits.
4. **Scene-text detection limitations**: Although scene-text detectors perform well on conventional benchmarks, they assume high resolution and clear text, and can fail in compromised maritime environments where IMO text suffers from rust, low contrast, and motion blur.

Table 1 systematically compares existing approaches (general detectors, small-object methods, scene-based text strategies, and multi-task learning with autoencoders) with our proposed framework across the key dimensions of small-object capability, scene text detection, spatial constraints, maritime focus, and handling of class imbalance.

In summary, the present research proposes a hybrid autoencoder–YOLO framework with a unique spatial regularisation technique that enables robust detection under extreme imbalance while maintaining spatial consistency.

## 3. Methodology

### 3.1. Design Explanation and Motivation

The two main obstacles to maritime IMO identification are (i) the wide gap between very small IMO numbers and massive ship objects, and (ii) the absence of spatial limitations in conventional object detectors. Small IMO events are often missed during training because standard one-stage and two-stage detectors optimise only classification and localisation losses. Because YOLOv8 balances detection accuracy, computational efficiency, and real-time inference speed, it was chosen as the baseline detector for marine security applications [11]. Numerous studies have shown YOLO’s usefulness for ship identification in a variety of maritime scenarios [46]. However, YOLOv8 alone does not leverage the fundamental spatial relationship between ships and IMO numbers or preserve precise image information.

To address these limitations, we develop a shared autoencoder with an additional reconstruction goal that encourages the encoder to learn richer feature representations. By making the network preserve high-frequency information that is crucial for identifying small objects, auxiliary tasks such as reconstruction have been shown to enhance feature learning in object detection [44]. This allows the network to retain high-frequency information, which is needed for identifying tiny IMO numbers. We also encode the prior that each IMO number must be placed inside a ship hull using a differentiable spatial containment loss, which improves localisation consistency without adding extra inference cost. An established method for improving prediction consistency and guaranteeing physical validity is to incorporate geometric constraints and spatial baselines into deep learning models [41]. Finally, a two-stage training approach stabilises optimisation by jointly optimising detection and spatial regularisation after first learning strong visual representations through reconstruction. Before combining more complex or inconsistent goals, two-stage training procedures are often used to first create a stable feature representation.

**Figure 2:**
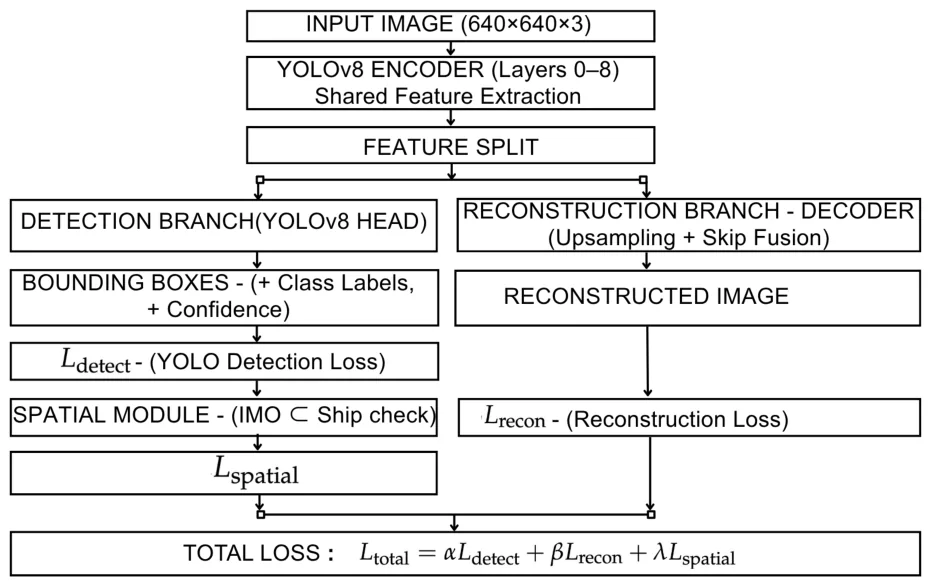
Workflow of the proposed hybrid YOLO–autoencoder framework with spatial regularization. The input image is processed by a YOLOv8 encoder. The encoder’s features are split into two branches: a detection head that predicts bounding boxes, class labels (SHIP/IMO), and confidence scores; a reconstruction decoder (incorporating upsampling and skip connections) then regenerates the input image. The detection outputs are input to a spatial regularization module that applies the maritime restriction (IMO numbers must reside within ship bounding boxes), resulting in a spatial loss $L_{\mathrm{spatial}}$. The overall training loss includes three components: detection loss $L_{\mathrm{detect}}$, reconstruction loss $L_{\mathrm{recon}}$, and spatial loss $L_{\mathrm{spatial}}$, weighted by hyperparameters α, β, and λ, respectively.

Figure 2 shows the proposed hybrid autoencoder–YOLO framework including spatial regularisation (the detailed architecture configuration is provided in Section S1 of the Supplementary Materials). The model processes an input image of dimensions 640 × 640 × 3 using a YOLOv8 backbone that functions as a shared encoder. Layers 0–8 generate structure-based feature representations via sequential spatial downsampling. After feature extraction, the network splits into two main routes. The first branch is the YOLO detection head, which predicts bounding boxes, class labels, and confidence scores. The second branch includes a reconstruction decoder that produces an image approximation of the input through a series of upsampling stages. A spatial restriction module is implemented on the detection outputs to ensure geometric accuracy between ships and IMO numbers. The model is trained inside a multi-task learning framework that sequentially optimises detection, reconstruction, and spatial consistency objectives. The comprehensive loss function is defined as follows:

(1) $$L_{\mathrm{total}}=\alpha L_{\mathrm{detect}}+\beta L_{\mathrm{recon}}+\lambda L_{\mathrm{spatial}}$$

where $L_{\mathrm{detect}}$ indicates the detection loss, $L_{\mathrm{recon}}$ represents the reconstruction loss, and $L_{\mathrm{spatial}}$ refers to the spatial regularization term. The ratios α, β, and λ control the relative importance of each component during training.

### 3.2. Detection Branch (YOLOv8 Head)

The detection branch is built on the YOLOv8 architecture and uses the backbone’s shared feature representation. The YOLOv8 backbone generates organised feature maps from a 640 × 640 × 3 input image through sequential downsampling, capturing both low-level and high-level meaning. The detection head receives these features and predicts item positions, classes, and confidence ratings in a single-stage approach. The detecting head generates bounding box coordinates (x, y, w, h), class probabilities, and objectness confidence for each expected situation. Unlike conventional two-stage detectors, YOLOv8 performs end-to-end prediction, enabling rapid real-time detection. This study improves the detection head for a dual-class marine context in which the model detects two categories: SHIP and IMO. The detection loss $L_{\mathrm{detect}}$ is calculated as a weighted combination of bounding box regression loss, classification loss, and objectness loss, in accordance with the conventional YOLOv8 framework (detailed hyperparameter settings for the detection branch, including learning rates, optimiser configuration, and loss weights, are provided in Section S2 of the Supplementary Materials):

(2) $$L_{\mathrm{detect}}=L_{\mathrm{box}}+L_{\mathrm{cls}}+L_{\mathrm{obj}}$$

where $L_{\mathrm{box}}$ penalizes localization errors, $L_{\mathrm{cls}}$ measures classification accuracy, and $L_{\mathrm{obj}}$ evaluates the confidence of object presence. This formulation allows for reliable detection of both large-scale objects (ships) and small targets with visual restrictions (IMO numbers). Still, significant class imbalance and spatial variability in marine environments make this approach insufficient, requiring reconstruction-based regularisation and spatial constraints within the suggested framework. As detection is the principal objective, we use α=1.0 in Equation (1); this weight maintains the detection loss at full strength, while spatial λ and reconstruction β serve as supporting components.

### 3.3. Reconstruction Branch

**Figure 3:**
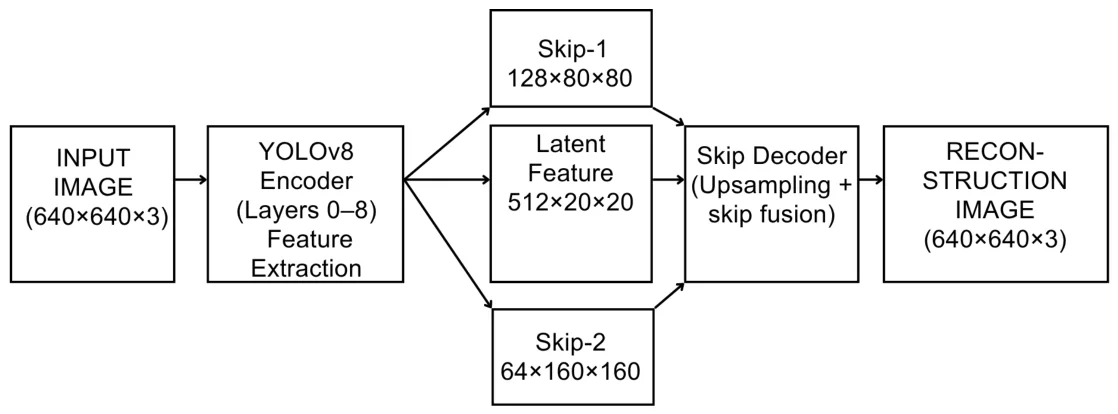
Workflow of the autoencoder used for reconstruction.

To improve feature learning at the boundaries under significant class imbalance and preserve complex spatial details, we incorporate an auxiliary reconstruction branch into the detection architecture. This branch utilises the YOLOv8 encoder (Section 3) and presents a SkipDecoder, a U-Net-style decoder that uses skip connections from intermediate encoder layers. Figure 3 shows the architecture of the proposed reconstruction branch, including the shared encoder, skip connections, and a reconstruction pipeline based on SkipDecoder (detailed layer configurations are provided in Section S1 of the Supplementary Materials).

The encoder produces a bottleneck feature map of size 512 × 20 × 20 and retains two skip features: one from layer 2 (64 × 160 × 160) and another from layer 4 (128 × 80 × 80). The SkipDecoder gradually improves the bottleneck through linear interpolation and convolutional blocks. During two upsampling stages, it concatenates the relevant skip features before additional convolution, allowing for recovery of the high-resolution spatial structures essential for detecting small IMO numbers.

The reconstruction loss $L_{\mathrm{recon}}$ encourages the decoded image $\hat{I}$ to match the input *I* at pixel, edge, and multi-scale levels. We use the L1 loss, defined as the mean absolute error between corresponding pixels:

(3) $$L1\left(\hat{I},I\right)=\frac{1}{N}\sum_{i=1}^{N}\left|{\hat{I}}_{i}-I_{i}\right|$$

where *N* is the total number of pixels (width × height × channels). L1 (mean absolute error) is preferred over L2 (mean squared error) for the loss due to its reduced sensitivity to outliers and capacity to provide sharper reconstructions, which is useful for maintaining small IMO text details [47]. The overall reconstruction loss is defined as follows:

(4) $$L_{\mathrm{recon}}=L1\left(\hat{I},I\right)+\lambda_{\mathrm{edge}}L1\left(E\left(\hat{I}\right),E\left(I\right)\right)+w\sum_{s\in \{2,4,8\}}L1\left(P_{s}\left(\hat{I}\right),P_{s}\left(I\right)\right)$$

where E(·) generates edge maps via the Sobel edge filters on the grayscale image, which helps to maintain the sharp boundaries of IMO numbers, while $P_{s}\left(\cdot \right)$ is the average pooling with a scale factor *s*, which enforces multi-scale consistency. We set $\lambda_{\mathrm{edge}}$ to 0.3 and *w* to 0.25 based on the sensitivity analysis in Folder 4 of the validation section (the experimental results are demonstrated in Section S2.5 of the Supplementary Materials). The chosen design with $\lambda_{\mathrm{edge}}=0.30$ and w=0.25 ensures a fair influence from the pixel-level, edge, and multi-scale reconstruction goals while avoiding auxiliary components from overshadowing the detection aim.

The edge term is supplementary; this weight promotes edge sharpness while preventing edge mistakes from overshadowing pixel-level integrity. With w=0.25, each multi-scale phrase is equally weighted (scales 2, 4, 8). Since all of the scale weights are consistent, we combine them into a single coefficient *w* outside the sum for clarity. The overall multi-scale contribution is low compared to the full-resolution L1 term.

This reconstruction branch acts as a hidden regulariser, forcing the shared encoder to preserve tiny details that would otherwise be ignored when optimising only for detection. In this way, the decoder branch enhances the representation of rare and tiny objects such as IMO numbers. We set β=1.0 to ensure that the reconstruction branch contributes equally to the overall loss, allowing the shared encoder to extract valuable visual features while keeping detection as the principal task (α=1.0).

### 3.4. Spatial Regularisation

To uphold the essential marine requirement that each IMO number must reside within a ship’s body, we propose a unique spatial regularisation loss. Unlike traditional methods that rely on ground-truth combinations, our loss function uses only the model’s predicted bounding boxes for ships and IMO numbers, instructing the network to respect spatial relationships without requiring specific administrative labels.

**Containment formulation:** Let ${\left\{B_{i}^{\mathrm{imo}}\right\}}_{i=1}^{N_{\mathrm{imo}}}$ be the set of predicted IMO boxes and ${\left\{B_{j}^{\mathrm{ship}}\right\}}_{j=1}^{N_{\mathrm{ship}}}$ the set of predicted ship boxes, where $N_{\mathrm{imo}}$ represents the quantity of expected IMO bounding boxes (IMO) and $N_{\mathrm{ship}}$ indicates the quantity of expected ship bounding boxes (ship) inside the current batch or image. The numbers vary between images and training cycles as the detector’s predictions improve; they are not static dataset counts. If no IMO is identified, then $N_{\mathrm{imo}}=0$; if no ship is found, then $N_{\mathrm{ship}}=0$.

For each predicted IMO *i*, we compute the maximum overlap ratio with any predicted ship:

(5) $$C_{i}=\max_{j}\frac{|B_{i}^{\mathrm{imo}}\cap B_{j}^{\mathrm{ship}}|}{|B_{i}^{\mathrm{imo}}|}$$

where |·| signifies the box area in pixels. The ratio measures how much of the IMO box is covered by a ship. Perfect containment yields $C_{i}=1$; a completely separate IMO results $C_{i}=0$.

**Spatial loss:** The spatial regularisation term below penalizes low containment scores.

(6) $$L_{\mathrm{spatial}}=\left\{\begin{array}{cc} 0 & \mathrm{if}\;N_{\mathrm{imo}}=0\\ 1 & \mathrm{if}\;N_{\mathrm{imo}}>0\;\mathrm{and}\;N_{\mathrm{ship}}=0\\ \frac{1}{N_{\mathrm{imo}}}\sum_{i=1}^{N_{\mathrm{imo}}}\left(1-C_{i}\right) & \mathrm{otherwise} \end{array}\right.$$

**Differentiability and the characteristics of gradients:** The conditional expression in Equation (6) incorporates a fixed penalty of 1 when $N_{\mathrm{imo}}$ exceeds 0 and $N_{\mathrm{ship}}$ equals 0. We recognise that the derivative of this fixed spatial loss component with respect to the network parameters is null in this branch; however, this does not suggest a null gradient for the comprehensive training objective. The spatial term is an additional regulariser, whereas the overall loss (Equation (1)) simultaneously encompasses the detection loss $L_{\mathrm{detect}}$ and the reconstruction loss $L_{\mathrm{recon}}$, both of which are continuously active and differentiable.

Specifically, when an IMO number exists but no vessel is anticipated, $L_{\mathrm{detect}}$ continues to penalise the unrecognised ship and provides the relevant gradient to improve the shared encoder and detection head. Once a ship prediction becomes available, the formulation transitions to the containment-based term in Equation (6), where the spatial regulariser becomes conditional on the prediction and provides gradients related to the respective spatial positions of the predicted IMO and ship boxes. Consequently, the fixed value of 1 should be treated as a maximum violation penalty for the spatial constraint rather than simply as the exclusive optimisation indicator for ship identification. This conditional formulation avoids using a spatial gradient when no ship reference is available, while the main detection goal remains capturing missed ship detections.

In the absence of an anticipated IMO, the loss is null (there is no restriction to apply). If IMOs are present but no ship is identified, the loss is set to 1 (maximum penalty), preventing this unavoidable situation. In the typical scenario where both IMO and ship boxes are present, the loss is calculated as the containment shortfall across all anticipated IMO boxes.

**Weighting (λ):** The spatial term is added to the total loss as $\lambda L_{\mathrm{spatial}}$. We use a predefined schedule for λ, starting at 0.01 and increasing gradually to 0.5 over 500 epochs. This gradual increase ensures that spatial regularisation acts as an auxiliary constraint early in training, allowing the detection and reconstruction tasks to establish meaningful features before the spatial prior is fully enforced. Additionally, an adaptive control mechanism reduces λ by 50% whenever $\frac{L_{\mathrm{spatial}}}{L_{\mathrm{detect}}}>0.5$, preventing the spatial term from destabilising training by dominating the total loss.

**Confidence threshold (τ) for box extraction:** To ensure that the spatial loss receives meaningful gradients despite weak early predictions, we apply a dynamic confidence threshold τ for extracting predicted boxes used in $L_{\mathrm{spatial}}$. We use a lower threshold (τ=0.02) during the first 20 epochs, allowing the spatial loss to operate even when detection confidences are low. After this warm-up period, we use a higher threshold (τ=0.05) to restrict spatial regularisation to more confident predictions, which reduces the influence of noisy detections. This approach makes the regularisation effective from the beginning of training while maintaining stability in later stages.

#### Limitations and Failure Scenarios of Spatial Regularisation

The suggested spatial regularisation loss successfully implements the physical restriction that IMO numbers should be confined within ship structures; however, it has several technical limitations that warrant examination.

1. **Overlapping ships:** When multiple ships overlap or seem to be in close range, the containment equation in Equation (6) could incorrectly assign an IMO number to an incorrect ship. This happens because the loss calculates the highest overlap ratio with any anticipated ship bounding box, ignoring distinctions between ship instances. In these cases, spatial loss can lead to false associations, reducing the localisation precision of both the ship and the IMO.
2. **Incorrect ship localisation:** The spatial deficiency is entirely dependent upon anticipated ship boundaries. If the detector provides incorrect ship bounding boxes (for instance, boxes that do not fully enclose the ship body), the containment scores lose reliability. These inaccuracies may arise throughout the optimisation procedure, as the spatial loss continues to impose containment based on potentially inaccurate ship references.
3. **Missing ship detections:** When the detector completely fails to identify a ship ($N_{\mathrm{ship}}=0$) despite the presence of IMO numbers, the spatial loss reduces to a fixed penalty of 1 (as elaborated in Section 3.4). Although this prevents the spatial loss from generating misleading gradients, it also implies that the batch offers no spatial direction, effectively reducing the regularising effect provided by the loss.
4. **Partial obstruction:** When IMO numbers are partially hidden by equipment, structural components, or other items on the ship deck, the containment loss may still be fulfilled (as the IMO box remains within the ship’s structure), but the reconstruction process may encounter difficulties in finding the hidden textual elements. This may result in overlooked IMO detections in highly obstructed situations.
5. **Multiple IMO labels:** Although uncommon, certain boats could have several IMO numbers (e.g., on either side of the hull). The current formulation does not explicitly address this, and could unfairly penalise IMO boxes that are appropriately positioned but not enclosed within a single ship box.
6. **Future improvements:** Various paths may address these shortcomings. Initially, instance-level association techniques could differentiate among various ships and IMO numbers in overlapping situations. Second, combining temporal data across video frames might help to recover missing detections and correct localisation inaccuracies. Third, adaptive weighting systems could adjust the spatial loss weight based on detection confidence, reducing the impact of low-confidence ship bounding boxes.

### 3.5. Training Strategy

The hybrid autoencoder–YOLO model uses a three-stage optimisation technique to apply reconstructive models effectively to the object detection problem.

#### 3.5.1. Stage I: Autoencoder Pretraining

Initially, the encoder (including the YOLOv8 backbone with skip connections) and SkipDecoder are trained separately as an image autoencoder. The encoder uses pretrained YOLOv8s weights, enabling robust initial feature extraction. Only the autoencoder parameters are optimised, using the Adam optimiser with a learning rate of $1\times 10^{-4}$ and weight decay of $1\times 10^{-5}$. The training objective is the reconstruction loss $L_{\mathrm{recon}}$ defined in Section 3.3. To improve robustness, training uses a mixed-data technique combining low-quality inputs (DAE-style corruption) and significantly augmented images, while remaining supervised with clean targets.

#### 3.5.2. Stage II: Hybrid Detection–Reconstruction Training

The pretrained encoder and decoder are integrated into a YOLOv8-based detection framework to support continuous learning of object detection and reconstruction. The model is designed for two classes (SHIP and IMO) and uses pretrained weights to initialise both the backbone and decoder modules, allowing reconstruction-aware features to transfer into the detection pipeline. Training occurs in two phases: in the initial phase (epochs 1–20), the encoder and decoder remain static while only the YOLO detection head is optimised, enabling stable detector adaptation without modifying pretrained representations; during this phase, the reconstruction branch is removed from the optimisation objective. During the second phase (epochs 21 and beyond), all components are unfrozen and trained together using a differential learning rate technique, with reduced learning rates for the encoder and decoder and higher rates for the detection head. The overall optimisation target is specified as follows:

(7) $$L_{\mathrm{total}}=\alpha L_{\mathrm{detect}}+\beta L_{\mathrm{recon}}$$

where $L_{\mathrm{detect}}$ and $L_{\mathrm{recon}}$ are defined in Section 3.1 and Section 3.3.

#### 3.5.3. Stage III: Spatial-Aware Hybrid Training

The spatial-aware extension extends the hybrid autoencoder–YOLO framework outlined in the next section using the FixedHybridYOLO architecture with a shared EncoderWithSkips backbone, SkipDecoder, and a dual-class detection head (SHIP and IMO). The model builds on the hybrid detection–reconstruction training above, ensuring identical feature representations for both reconstruction and detection tasks. Training follows an identical two-phase technique as the hybrid model. During Phase 1 (epochs 1–20), the encoder and decoder remain static while only the detecting head is optimised, with reconstruction disabled. In Phase 2 (epoch 21 and beyond), all components are unfrozen and trained continuously with differential learning rates. The main difference in this spatial-aware contrast comes in the training objective. We add a spatial consistency term to the optimisation process alongside the detection and reconstruction losses. This term uses the compute_losses_with_spatial function (Equation (1)), which enhances the conventional detection loss framework by adding spatial constraints on predicted bounding boxes.

The spatial loss applies only to predictions and is scaled by a factor λ, which increases progressively during training to support stable optimisation. This requires geometric agreement between anticipated ships and IMO numbers without requiring extra annotations.

## 4. Experimental Setup

### 4.1. Dataset

The collection has 297 labelled images, split into a designated test set of 47 images and a 5-fold cross-validation set of 250 images (200 for training and 50 for validation in each fold). We assess a maritime object detection dataset which includes 297 annotated images categorised into two classes: (SHIP and IMO). We set aside a fixed hold-out test set of 47 images before any cross-validation, and it remains unused during both training and validation. The remaining 250 images are allocated to a 5-fold cross-validation strategy. In each fold, 200 images are used for training and 50 for validation, ensuring that each image appears exactly once in the validation set across the five folds. In each fold, the split allocates 80% (200 images) for training and 20% (50 images) for validation, following standard cross-validation practice. The static test set (47 images) is kept separate and utilised exclusively for the conclusive assessment. Images are resized to 640 × 640 using letterbox preprocessing [48], which preserves the aspect ratio by adding padding and maintains geometric consistency across the dataset.

**Data collection and construction:** We gathered images from the publicly accessible maritime ship database on the Baltic Shipping website to create the dataset [49]. The absence of high-quality ship images with clear IMO numbers made image collection difficult. Various lighting conditions (low light, overcast, sunny, and night), weather conditions (clear, foggy, and cloudy), viewing angles (front, side, and angles), and distances (close-up, medium-range, and far-away ships) are included in the collection of images. IMO marks on ships vary significantly in their size, font style, positioning, contrast, and visibility due to issues including body colour, surface damage, rust, obstruction by equipment or structural elements, and image resolution. In order to ensure thorough coverage, the collection includes ships of all kinds (cargo, tanker, fishing, and container ships), with IMO numbers ranging from very small and low contrast to clearly visible. Section S4 of the Supplementary Materials provides a more detailed description of the dataset collection and construction process, including the two-stage screening and annotation protocol. Supplementary Table S13 provides a detailed breakdown of the dataset composition across lighting conditions, weather conditions, and ship types. We used Roboflow [50], an online annotation tool, to pre-label every image with bounding boxes around SHIP instances and IMO number regions. Each IMO bounding box carefully wrapped the digit sequence without covering nearby ship area, and we personally reviewed and refined the annotations to ensure accuracy. Quality assurance checks confirmed annotation consistency.

**Figure 4:**
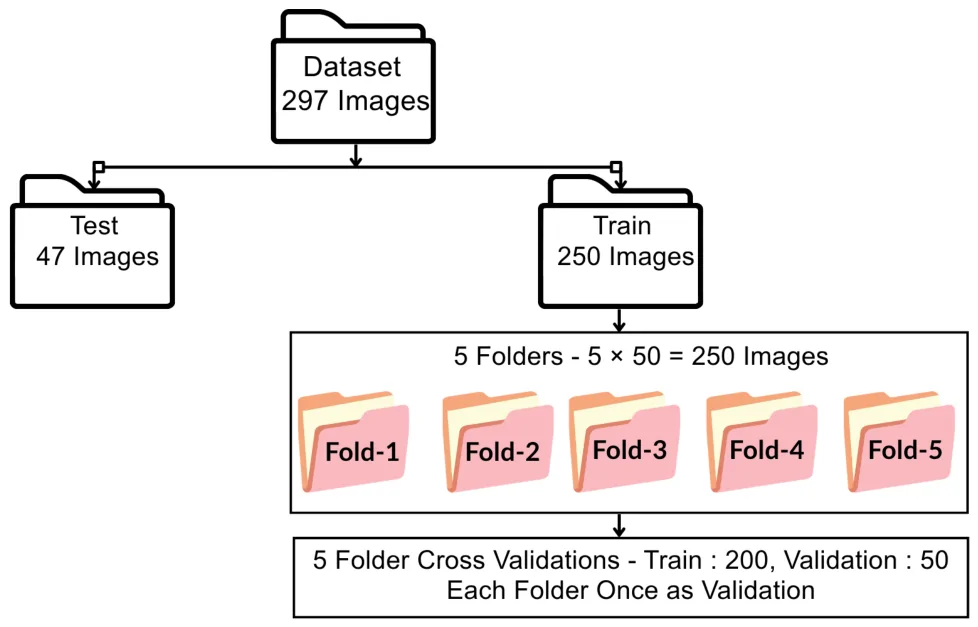
The dataset split and cross-validation strategy.

Figure 4 shows the dataset partitioning strategy: 250 images are used in a 5-fold cross-validation scheme with 200 training images and 50 validation images per fold, with a fixed hold-out test set of 47 images reserved solely for the final evaluation.

**Related datasets:** Comparable datasets. Few datasets offer the precise combination of ship and IMO annotations needed for this investigation, despite multiple datasets for ship and identification number detection. To resolve visual confusion from ship alignment, the NavShip-5V (Navigation-oriented Ship 5-Viewpoints) dataset is a novel multi-view dataset annotated with a function-oriented protocol for autonomous IMO inspection by UAVs [51]. The Roboflow Universe Ship IMO Number Detection 2 dataset is openly accessible for computer vision tasks and has two classes (SHIP and IMO) [52]. With 1472 ship identities and 88,862 images for ship identification, the SLPDR (Ship License Plate Detection and Recognition) collection is the first significant benchmark [53]. However, these datasets lack the precise combination of ship-level and IMO-level annotations needed for our analysis, are not publicly available, or were created for alternative goals (such as viewpoint estimation). By offering a carefully selected set of 297 annotated images with both SHIP and IMO bounding boxes, our dataset fills this gap. We created it specifically to assess object detection models on the difficult problem of small-text IMO recognition in real maritime contexts.

**Autoencoder pretraining data:** During the reconstruction pretraining phase (Stage I in Section 3.5), each training sample includes an input–target pair. The input is produced using either (i) degradation-based corruption (e.g., Gaussian noise, blurring), or (ii) robust YOLO-style augmentation (mosaic, mixup, HSV jitter, flips, affine transformations). The objective is consistently the clean letterboxed image. A mixed sampling technique uses roughly 50% degradation-based samples and 50% YOLO-style samples. Validation uses only raw letterbox images without enhancement.

**Hybrid detection–reconstruction data:** In the joint training phase (Stage II and III in Section 3.5), each sample includes an image along with its bounding box annotations (SHIP and IMO). Training images undergo augmentation with YOLO-style changes, including HSV jitter, horizontal flipping, random affine transformations, mosaic, and mixup techniques. The identical input image serves as the reconstruction goal, ensuring consistency between detection and reconstruction tasks. The validation data use only letterbox resizing, with no enhancement.

**Dataset limitations and overfitting mitigation:** We recognise that 297 labelled images provide a rather limited dataset for training deep neural networks, especially considering the supplementary reconstruction and regularisation branches in our architecture. We used several techniques to mitigate overfitting and ensure model generalisation. Initially, we employed transfer learning by initialising the YOLOv8 encoder with weights pretrained on COCO, which provides robust feature initialisation and reduces the need for extensive domain-specific datasets. Second, we applied comprehensive data augmentation throughout training, including mosaic, mixup, HSV jitter, horizontal flipping, random affine transformations, and degradation-induced corruption (Gaussian noise, blurring). The enhancement process is as follows: LetterBox combined with mosaic yields MixUp, followed by HSV adjustments, flipping, and affine transformations for training, while validation uses only LetterBox resizing. Third, we used 5-fold cross-validation with a specified hold-out test set (47 photos), ensuring that each image is used for validation exactly once across the five folds and delivering a reliable assessment of model performance. Fourth, the reconstruction component functions as a regulariser, requiring the shared encoder to maintain detailed characteristics and mitigate overfitting to the standard ship category. Fifth, we included weight decay (0.0005) and a cosine annealing learning rate scheduler to enhance training stability and mitigate overfitting. Despite these initiatives, we recognise that the dataset’s limited size restricts thorough assessment across many marine contexts, and validation on more extensive public datasets is an essential path for future research.

### 4.2. Baseline Models and Data Compatibility

The initial YOLOv8 method serves as a performance benchmark with standard object detection systems. This baseline focuses only on optimising direct detection, without supplementary tasks or spatial reasoning. To compare with standard models (EfficientDet, Faster R-CNN, RetinaNet, and DETR) we use identical 5-fold cross-validation splits and the same hold-out test set utilised in our hybrid model. We transform the original YOLO-format annotations into COCO JSON for Faster R-CNN, RetinaNet, and DETR and into TFRecord for EfficientDet. We train all baseline models with their default hyperparameters as specified in their official implementations (e.g., Detectron2 for Faster R-CNN and RetinaNet, Hugging Face for DETR, and the official EfficientDet repository), with the only real modification being the number of output classes (two: SHIP and IMO). We make no supplementary task-specific adjustments to ensure a fair and reliable comparison.

- **YOLOv8s** [13]—a popular single-stage anchor-free detector.
- **RetinaNet** [43]—a single-stage detector using focal loss to address class imbalance.
- **Faster R-CNN** [45]—a two-stage region-based detector.
- **DETR** [54]—a transformer-based end-to-end object detector.
- **EfficientDet** [55]—a single-stage detector using BiFPN and compound scaling for efficient and scalable feature fusion.

All models initially start with their respective standard COCO-pretrained backbones (CSPDarknet for YOLOv8s, ResNet50 for RetinaNet and Faster R-CNN, EfficientNet-B0 for EfficientDet, and the standard backbone for DETR) and are eventually fine-tuned on our dataset for 500 epochs.

### 4.3. Experimental Setup

We performed all experiments on an NVIDIA DGX A100 GPU (NVIDIA, Santa Clara, CA, USA) system running a Jupyter environment. The setup used Python 3.11 and an NVIDIA A100-SXM4-80GB GPU with CUDA 12.1 acceleration. We used PyTorch 2.2.2 with CUDA 12.1 support to implement the models, along with additional libraries such as Ultralytics YOLO, OpenCV 4.10.0.82, NumPy 1.26.4, Albumentations 1.3.1 and Pydantic 1.10.13. We assessed inference speed on the same NVIDIA A100 GPU with a batch size of 1 to investigate computational efficiency and ensure consistent delayed measurement across all comparison models. We trained all models at an input resolution of 640 × 640 pixels with a batch size of 8. We ran the autoencoder pretraining phase for 500 epochs to enable robust reconstruction learning. We then trained the suggested hybrid and spatial-aware models for 500 epochs using the cross-validation technique outlined in Section 4.1.

We trained standard detection models for up to 500 epochs for baseline comparison to ensure sufficient convergence within their specific training parameters. A YOLOv8-small model (YOLOv8s.pt) was trained. We used the Adam optimiser with a learning rate of 0.001 and conventional data augmentation to improve prediction and reduce overfitting. The loss function followed YOLOv8s standard configuration, assigning weights of 0.5 for classification, 7.5 for bounding box regression, and 1.5 for distribution-focused loss. During inference, we filtered predicted bounding boxes with a confidence threshold of 0.50 and an IoU threshold of 0.45. We selected the model with the lowest validation detection loss.

### 4.4. Evaluation Metrics

#### 4.4.1. Detection Metrics

Detection performance is evaluated using precision, recall, and F1-score [56], in addition to mean average precision across intersection over union (IoU) thresholds ranging from 0.5 to 0.95 (mAP50, mAP50-95) [57]. These metrics assess the model’s precision in identifying and localising items (SHIP and IMO) according to different overlap thresholds.

Precision (*P*) indicates the ratio of accurately identified positive detections to the total estimated positives, and is calculated by

(8) $$\mathrm{Precision}=\frac{TP}{TP+FP},$$

where TP represents the number of true positives and FP the number of false positives.

Recall (*R*) evaluates the ratio of accurately identified positive cases to the total number of genuine positives, and is calculated by

(9) $$\mathrm{Recall}=\frac{TP}{TP+FN},$$

where FN represents the number of false negatives.

F1-score is the harmonic mean of precision and recall, offering a balanced measure of detection accuracy. It is calculated by

(10) $$F1=2\cdot \frac{\mathrm{Precision}\cdot \mathrm{Recall}}{\mathrm{Precision}+\mathrm{Recall}}.$$

Mean average precision (mAP50, mAP50-95) is the average of the average precision values calculated across multiple IoU thresholds from 0.5 to 0.95 (step 0.05) for all object classes. This captures both moderate (IoU = 0.5) and strict (IoU = 0.95) localisation criteria.

#### 4.4.2. Reconstruction Metrics

The peak signal-to-noise ratio (PSNR) measures the reconstruction quality of images by comparing the rebuilt output with the ground truth. It is calculated as

(11) $$\mathrm{PSNR}=10\cdot \log_{10}\left(\frac{{\mathrm{MAX}}_{I}^{2}}{\mathrm{MSE}}\right),$$

where ${\mathrm{MAX}}_{I}$ is the maximum possible pixel value (e.g., 255 for 8-bit images) and MSE is the mean squared error between the reconstructed image and the ground truth image [58].

The structural similarity index (SSIM) measures perceptual image quality by evaluating brightness, contrast, and structure [58]. It is calculated as

(12) $$\mathrm{SSIM}\left(x,y\right)=\frac{\left(2\mu_{x}\mu_{y}+C_{1}\right)\left(2\sigma_{xy}+C_{2}\right)}{\left(\mu_{x}^{2}+\mu_{y}^{2}+C_{1}\right)\left(\sigma_{x}^{2}+\sigma_{y}^{2}+C_{2}\right)},$$

where $\mu_{x},\mu_{y}$ are mean intensities, $\sigma_{x},\sigma_{y}$ are standard deviations, $\sigma_{xy}$ is the cross-covariance, and $C_{1},C_{2}$ are small constants for numerical stability.

The learned perceptual image patch similarity (LPIPS) is a deep learning-based metric that computes the $\ell_{2}$ (Euclidean) distance between deep features of two images extracted from a pretrained network (e.g., VGG [59]), defined as follows:

(13) $${∥u-v∥}_{2}=\sqrt{\sum_{i=1}^{N}{\left(u_{i}-v_{i}\right)}^{2}}$$

where *u* and *v* represent the respective feature vectors from the two images. Reduced LPIPS values signify more perceptual similarity between the reconstructed and target images.

## 5. Experimental Results

We performed a sensitivity analysis of the reconstruction loss parameters ($\lambda_{\mathrm{edge}}$ and *w*) on Folder 4 to validate the chosen values. The findings, detailed in Supplementary Table S7, validate that the selected configuration achieves an equitable compromise among pixel-level accuracy, edge preservation, and multi-scale consistency.

### 5.1. Reconstruction Performance in Autoencoder

**Figure 5:**
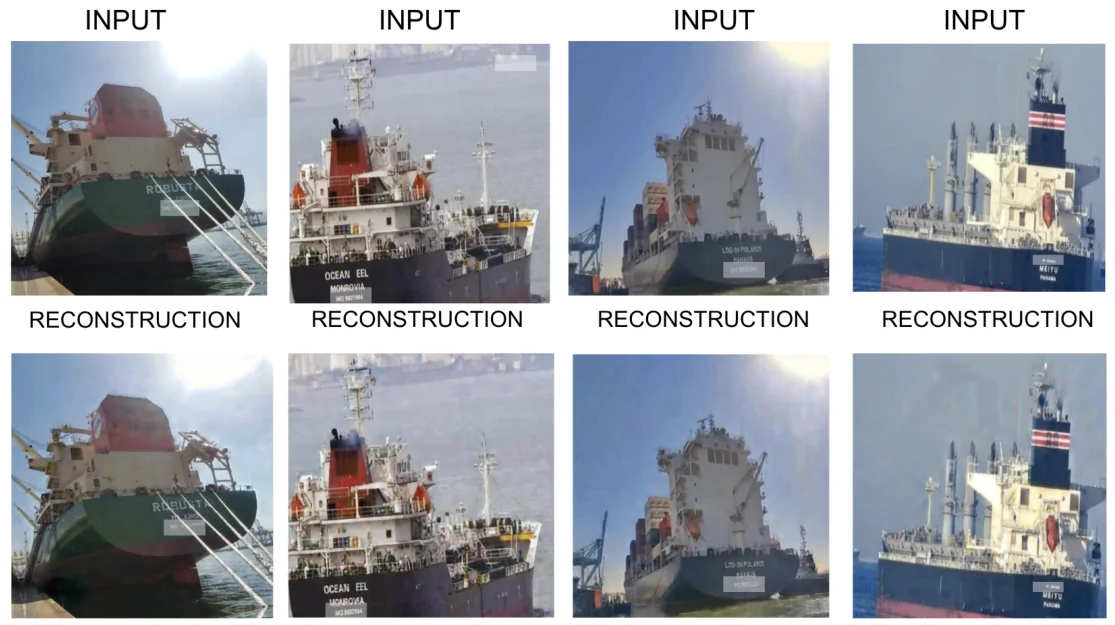
Reconstruction results on ship datasets, showing input images and reconstructed outputs. White boxes indicate the IMO numbers, which are shown in zoomed detail in Figure 6.

To assess the autoencoder’s ability to retain precise information, particularly small IMO text, we evaluate its reconstruction performance on the marine ship dataset. Figure 5 presents representative examples: the original input images (top) and their matching reconstructed outputs (bottom). The reconstructed images qualitatively preserve the main structure and most features; however, some fine text (IMO numbers) appears slightly flat, as expected for a typical autoencoder with a bottleneck.

**Figure 6:**
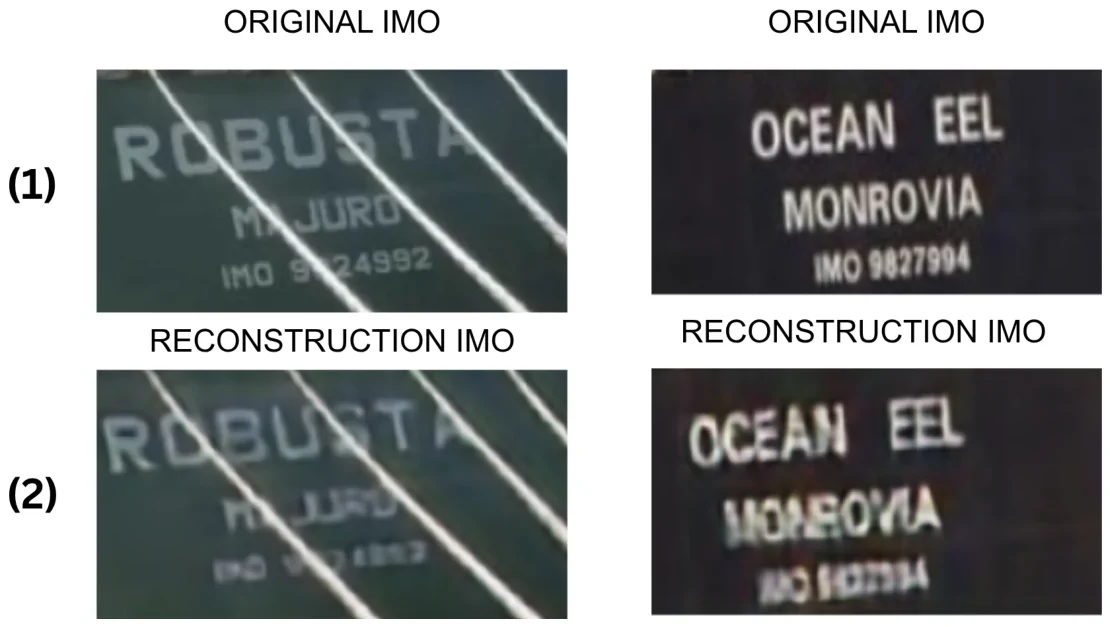
Cropped IMO regions: (**1**) original vs. (**2**) reconstructed.

To enhance the visualisation of this effect, Figure 6 presents cropped areas surrounding the IMO text for two instances, combining the original (1) and reconstructed (2) areas. The text remains readable but loses sharpness, characteristic of a bottleneck autoencoder that compresses information. In this case, the encoder effectively extracts selective features, as shown by the downstream detection performance.

To confirm that the reconstruction branch improves IMO-related feature channels despite visually indistinct reconstructions, we use Grad-CAM [60] to show the spatial areas that most strongly activate the encoder for IMO identification. Although SHAP analysis has been investigated for understanding object detection via frameworks such as DetDSHAP [61] and FSOD [62], Grad-CAM offers a computationally efficient and widely accepted option for visualising feature significance in CNN-based detectors.

**Figure 7:**
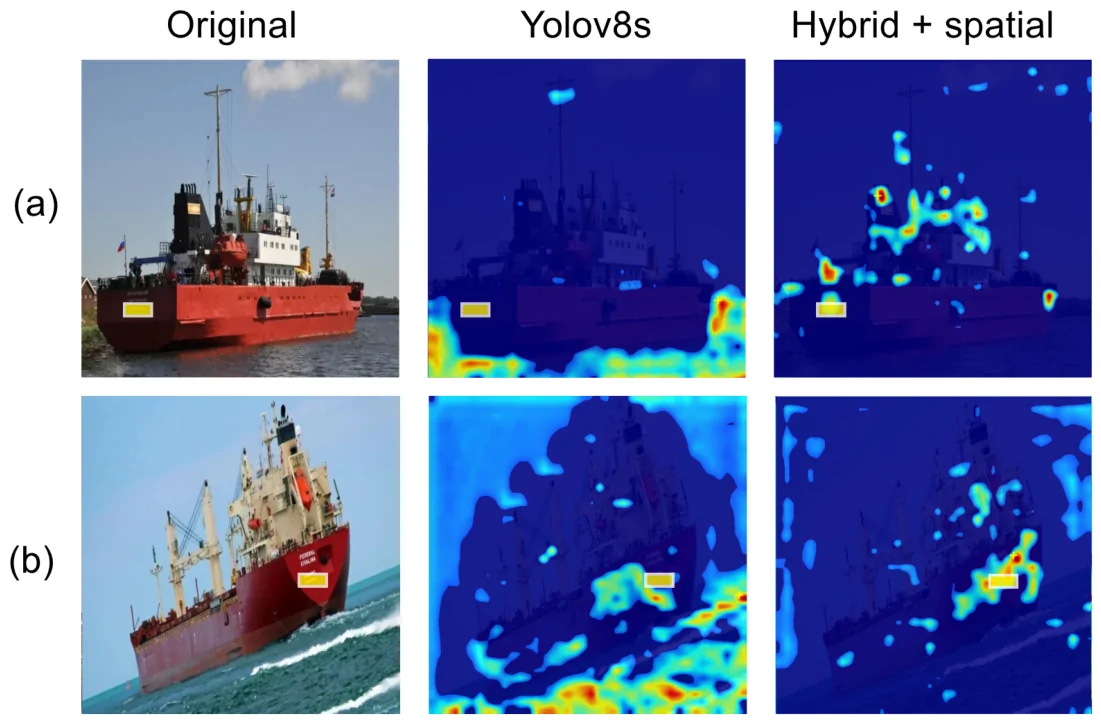
Grad-CAM visualisation comparing encoder activations with and without reconstruction supervision. (**a**,**b**) show two different test images. For each image: **Left**: input images with ground-truth IMO numbers highlighted (boxes); **Middle**: Grad-CAM heatmaps from baseline YOLOv8s (no reconstruction); **Right**: Grad-CAM heatmaps from the proposed Hybrid+Spatial model. The baseline exhibits weak and diffuse activation over IMO text regions, while our model produces sharply focused high-intensity activations precisely aligned with the IMO digit sequences. This confirms that reconstruction supervision selectively enhances IMO-relevant feature channels despite blurry reconstructions. (The color scale indicates activation intensity, with yellow/red representing high activation and blue representing low activation.)

Figure 7 shows Grad-CAM heatmaps generated by the baseline YOLOv8s and our full Hybrid+Spatial model on the same test image.

The baseline YOLOv8s shows uneven and weak activation over the IMO text numbers, with the model often attending to irrelevant background areas or ship body edges. As a result, our Hybrid+Spatial model generates clearly targeted high-intensity activations that are accurately synchronised with the IMO number sequences. This indicates that although the reconstruction branch generates visually identical reconstructions (Figure 6), it forces the shared encoder to assign greater attention weights to the significant high-frequency features of IMO text. These results directly validate our claim of ‘selective feature extraction’ and confirm that reconstruction supervision specifically enhances IMO-relevant feature channels.

We evaluate quantitative performance using conventional image quality criteria, including PSNR, SSIM, and LPIPS [57]. Table 2 shows the autoencoder’s reconstruction efficiency across both datasets.

These values show high reconstruction accuracy. The model achieves a PSNR of 26.18 dB, indicating excellent signal-to-noise quality in the reconstructed images. The SSIM of approximately 0.87 indicates robust structural preservation; however, the LPIPS of 0.24 indicates moderate perceptual disparity, primarily due to blurring in high-frequency text areas. Despite this, the encoder successfully extracts selective features, as shown by the downstream detection performance (Section 5.1). The reconstruction metrics indicate that the autoencoder functions effectively as a feature extractor for the hybrid detector in spite of imperfect text reconstruction.

### 5.2. Comparison with Benchmark Detectors

To evaluate the inherent challenge of IMO detection and establish a fair benchmark, we compare popular general-purpose object detectors on the same marine ship dataset. All models are initialised with their respective standard COCO-pretrained weights, fine-tuned on the training set (200 images), and tested on the test set (47 images, 102 instances) with identical preprocessing (640 × 640, letterbox) and evaluation criteria (confidence threshold 0.50, IoU 0.45).

**Figure 8:**
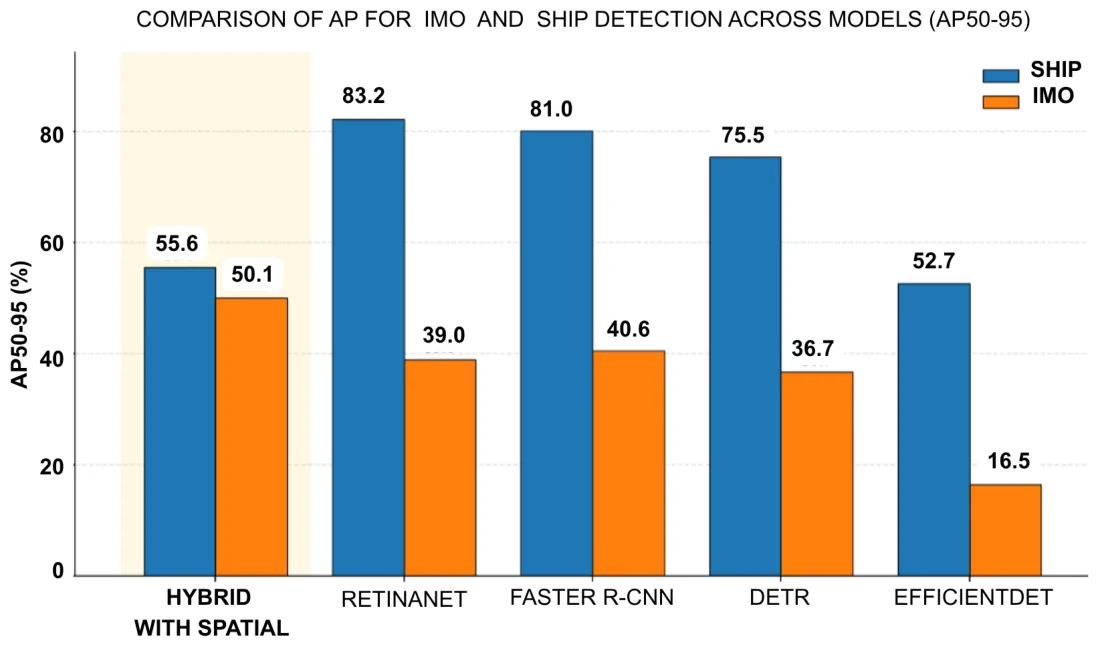
Comparison of AP50-95 for ship and IMO detection across baseline detectors and the proposed Hybrid+Spatial model.

Figure 8 shows the per-class AP50-95 as a grouped bar chart. All detectors show decent performance in ship identification, with AP50-95 values ranging from 75.5% to 82.3%, indicating that large ships are quite straightforward to localise.

In contrast, IMO detection presents considerable difficulties. Figure 8 compares the proposed Hybrid+Spatial model with several cutting-edge detectors for ship and IMO detection. The suggested model achieved a maximum IMO AP50-95 of 50.1%, beating RetinaNet (39.0%), Faster R-CNN (40.6%), DETR (36.7%), and EfficientDet (16.5%). This outcome shows the impact of combining reconstruction-guided feature learning with spatial regularisation for identifying small and low-contrast IMO numbers. Conventional detectors perform much better for ship detection, as shown by RetinaNet’s 82.3%, with other approaches showing similarly high performance. The proposed model achieves 55.6% for ship detection, indicating a calculated compromise prioritising enhanced IMO recognition, which is the principal aim of this study. EfficientDet shows the worst IMO performance despite achieving 52.7% for ship identification, indicating that its feature scaling and fusion technique is insufficient for extremely small text-based targets. The large performance gap between ship and IMO detection highlights the challenges of small-object text detection in marine images. At the same time, the proposed method shows that task-specific feature learning and geometric constraints can significantly improve IMO localisation accuracy.

**Figure 9:**
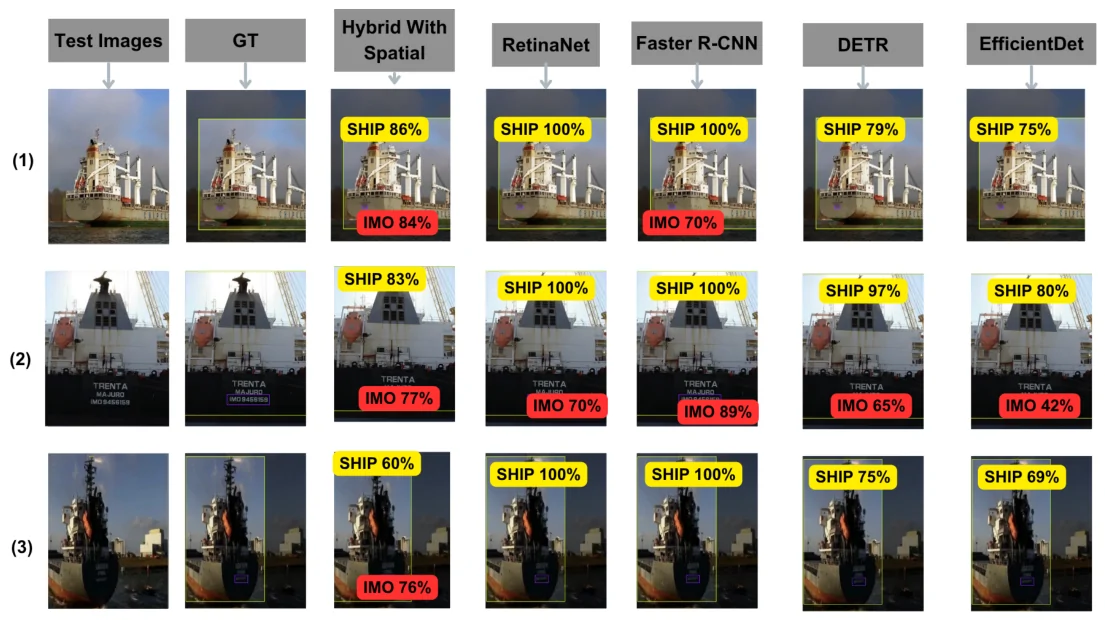
Sample prediction results with confidence scores from Hybrid+Spatial, RetinaNet, Faster R-CNN, DETR, and EfficientDet on three test images on three test images (**1**), (**2**), and (**3**).

Figure 9 shows sample prediction results from each detector on three test images.

The suggested Hybrid+Spatial model performs consistently on both tasks, with detection confidence on ships reaching 60%, 83%, and 86% across the three test images and IMO detection confidence reaching 84%, 77%, and 76%. In particular, RetinaNet and Faster R-CNN fail to detect the IMO in at least one image each for images 1 and 3, where the IMO numbers are very small and hard to see. RetinaNet misses IMO in images 1 and 3, while Faster R-CNN misses IMO in image 3. In images 1 and 3, both DETR and EfficientDet cannot identify an IMO, and EfficientDet achieves only 42% confidence on the single visible IMO in image 2. By contrast, the Hybrid+Spatial model effectively recognises the IMO in all three images, including the difficult tiny IMO samples, demonstrating the value of spatial regularisation and reconstruction-guided feature learning for small text detection.

Table 3 compares the speed of all assessed detectors on an NVIDIA A100 GPU with a batch size of one. The Hybrid+Spatial model shows the quickest inference time at 8.0 ms per image, slightly beating YOLOv8s at 10.0 ms. However, RetinaNet (84.6 ms), Faster R-CNN (98.8 ms), and DETR (69.9 ms) require much longer processing times, while EfficientDet balances precision and speed, achieving 17.5 ms per image. These results show that the suggested architecture maintains real-time performance while delivering enhanced IMO detection accuracy.

### 5.3. Comparison with Baseline YOLOv8s and Ablation Study of the Hybrid Autoencoder Module

First, we assess the impact of the shared encoder and the reconstruction task without the spatial containment loss. Table 4 contrasts three models: the baseline YOLOv8s, our hybrid model excluding spatial loss, and the complete hybrid incorporating spatial loss. All models are assessed using the identical test set of 47 images, 48 IMO numbers, and 54 ship instances. All models use the same evaluation protocol consisting of a confidence threshold of 0.50 and an IoU threshold of 0.45. We evaluate inference speed on an NVIDIA A100 GPU with a batch size of one.

#### 5.3.1. Effect of the Shared Encoder

The hybrid model significantly improves IMO detection, which is the principal aim of this study, namely, accurate detection of small objects. In comparison to YOLOv8s, AP50 rises from 85.3% to 89.9% (+4.6%), while the more strict AP50-95 improves from 42.3% to 46.8% (+4.5%). Precision, recall, and F1 increase from 91.3% to 93.6%, 87.5% to 91.7%, and 89.4% to 92.6%, respectively. These consistent improvements suggest that the shared autoencoder boosts lightweight feature representation, particularly helping to recognise small and small IMO text.

In contrast, ship detection shows a trade-off: AP50 declines from 92.5% to 72.2% and recall drops from 92.6% to 79.6%, while precision remains flawless at 100%. This indicates that the model becomes more selective, occasionally missing ship cases in favour of a more robust emphasis on IMO. However, the total F1-score remains high at 93.8%, although overall mAP50 declines from 88.9% to 81.3% due to dataset imbalance between ship and IMO events. Finally, inference speed improves from 10.0 ms to 8.0 ms per image, possibly because the shared encoder reduces duplicate processing, showing that performance improves without sacrificing efficiency.

#### 5.3.2. Effect of Spatial Containment Loss

To augment IMO localisation, we introduce a spatial containment loss that penalises anticipated IMO boxes positioned outside the ship hulls. The loss is calculated as the average of 1 minus max_containment over all IMO boxes, where containment refers to the intersection over union (IoU) between an IMO box and the optimal ship box. This term is multiplied by a small but progressively increasing weight λ (starting at 0.01) to act as a minor regulariser without overshadowing the detection loss.

The use of spatial loss enhances the hybrid model, especially for IMO detection, which is the main goal. In comparison to the hybrid model without spatial constraints, AP50 achieves a minor rise from 89.9% to 90.7% (+0.8%), while AP50-95 shows a significant enhancement from 46.8% to 50.1% (+3.3%), indicating significantly better localisation accuracy and enhanced alignment of predicted bounding boxes with IMO text regions. Precision rises from 93.6% to 97.8% (+4.2%), indicating a significant reduction in false positive IMO detections, while recall remains constant at 91.7%, confirming that the quantity of accurately identified actual IMO numbers is unchanged. Thus, the F1-score achieves 94.6%, indicating the optimal overall balance across all configurations.

At the same time, ship detection shows a negligible trade-off, with AP50 remaining at 72.2%, recall decreasing marginally from 79.6% to 77.8%, and precision declining from 100% to 97.7%, indicating the model’s improved priority for spatial consistency between the ship and IMO. Despite this, the overall mAP50-95 improves only slightly, from 52.4% to 52.8%, while overall precision remains flawless at 100%. Inference speed remains constant at roughly 8.0 ms per image, as the spatial loss is applied only during training and adds no processing burden during testing. As a result, the comprehensive hybrid model with spatial regularisation provides optimal IMO detection accuracy while ensuring fast inference, forming the foundation of our suggested approach.

#### 5.3.3. Predicted Examples

**Figure 10:**
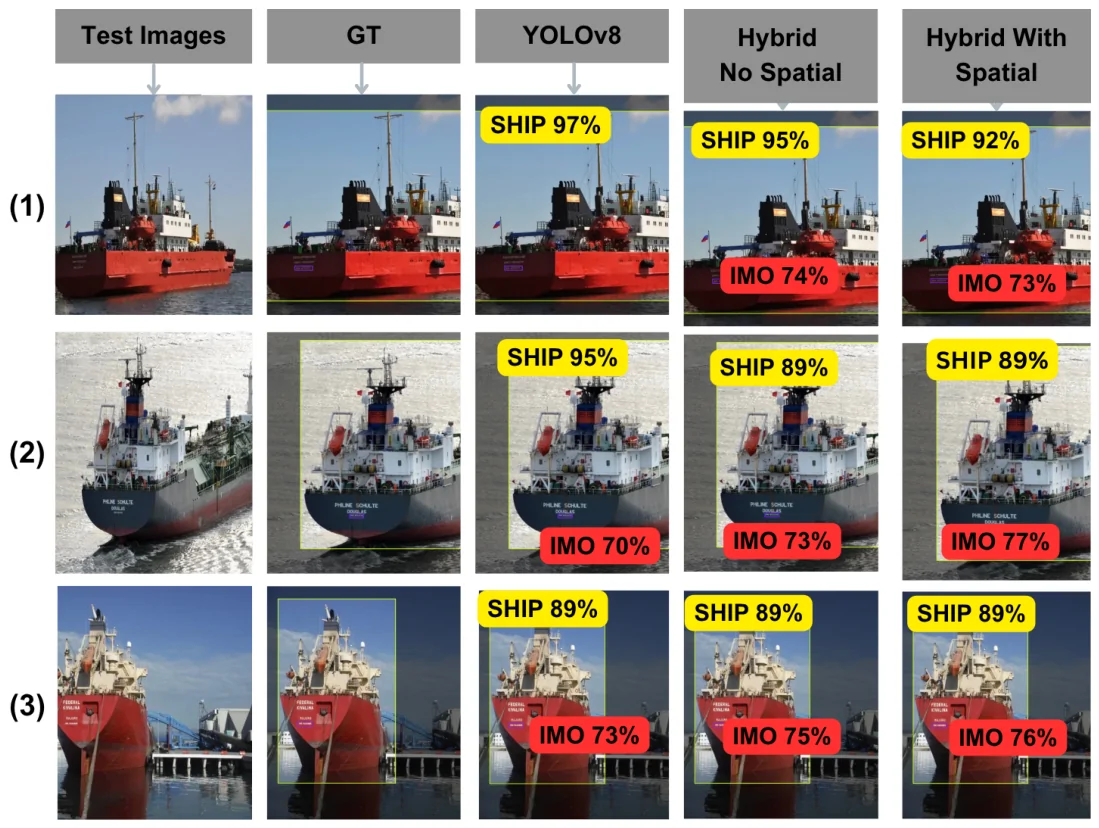
Absolute detection outcomes on three test images (**1**), (**2**), and (**3**). GT indicates ground truth. For each model, ship confidence is displayed at the top and IMO confidence at the bottom (when applicable). Red boxes signify IMO detections, while yellow boxes indicate ship detections.

Figure 10 compares the detection outputs of YOLOv8, the hybrid model without spatial loss (Hybrid Autoencoder–YOLO), and the comprehensive hybrid model with spatial regularisation (Hybrid AE–YOLO + Spatial) across three typical test images. Each shows the ground-truth (GT) bounding box for the ship, along with the predicted ship and IMO confidence levels.

YOLOv8 accurately identifies ships in all three images with high confidence (97%, 95%, and 89%). However, IMO detection is less reliable: no IMO prediction is generated in the first image and IMO numbers are identified only in the second and third images, with confidence scores of 70% and 73%, respectively. This suggests the baseline detector struggles to consistently localise small text-based targets even while maintaining robust ship identification performance.

The hybrid autoencoder–YOLO model without spatial loss enhances IMO identification by accurately identifying IMO numbers in all three images, giving confidence scores of 74%, 73%, and 75%, respectively. Ship detection remains stable, with confidence scores of 95%, 89%, and 89%. The findings indicate that reconstruction-guided feature learning improves selective representations for difficult small-object recognition.

The complete model (Hybrid AE–YOLO + Spatial) shows the most reliable qualitative performance. IMO numbers are identified in all three images with confidence values of 73%, 77%, and 76%, indicating greater confidence and stability than the non-spatial hybrid model. Ship detection remains dependable at 92%, 89%, and 89%. The spatial loss improves localisation by ensuring tighter alignment of IMO predictions within the ship body region, minimising visually evident false positives and refining bounding box placement while maintaining detection accuracy.

## 6. Discussion and Conclusions

### 6.1. Discussion

The experimental findings indicate that the proposed hybrid autoencoder–YOLO architecture effectively addresses two critical issues in maritime object detection: severe class imbalance, and spatial inconsistency between ship bodies and IMO text placement. Rather than repeating the numerical enhancements detailed in the Results section, this discussion emphasises the reasons behind the reported numerical improvements, the role of each component, and their practical impacts.

A key contribution of the system is reconstruction-based pretraining via autoencoder. Unlike conventional detection pipelines that optimise only classification and localisation losses, the reconstruction objective forces the shared encoder to preserve detailed spatial information across the feature structure. This is especially crucial for IMO numbers, which are small, low-frequency, and often visually impaired. Table 4 shows that the hybrid model without spatial loss improves IMO AP50-95 from 42.3% (YOLOv8s) to 46.8% (+4.5 percentage points) and increases IMO recall from 87.5% to 91.7%, validating that the reconstruction branch improves feature representation for rare objects. Although reconstructed outputs may reduce high-frequency text areas, the encoder retains the essential classification signals needed for further identification. This shows a steady improvement in IMO detection efficiency with the introduction of the reconstruction branch, confirming that auxiliary self-supervision augments representation learning without requiring additional observations.

**Understanding the performance gains:** The numerical improvements in Table 4 stem from two main factors. First, the reconstruction branch forces the shared encoder to preserve high-frequency spatial details that conventional detection frameworks often overlook. This is particularly beneficial for IMO numbers, which represent only 1.2% of labelled items and average only 17 pixels in height. The reconstruction objective serves as a self-supervised regulariser, encouraging the encoder to preserve the complex characteristics essential for identifying IMO numbers against background textures such as rust, damage, or ship marks. Second, the spatial regularisation loss aims to reduce false positives by penalising IMO detections located outside the boundaries of ship bodies. Table 4 shows that IMO precision increases from 91.3% (YOLOv8s) to 97.8% (Hybrid+Spatial), indicating a 6.5 percentage point decrease in false positives. This confirms that the spatial limitation successfully removes physically inconsistent detections, such as IMO numbers appearing on the marine surface or on nearby structures, without requiring more annotated data.

The combination of reconstruction-guided feature maintenance and spatial consistency regulation effectively addresses the dual concerns of class imbalance and spatial inconsistency. Ablation studies on bottleneck dimensions, spatial loss formulations, and training methods validate the architectural and training design decisions (see Supplementary Tables S8–S12). The findings validate that the existing setup offers the optimal balance for maritime IMO detection.

**Trade-off between ship detection and effectiveness of spatial loss:** The observed reduction in ship AP50-95 (77.0% → 55.6%) raises a key question: if the spatial decrease relies on anticipated ship boxes, how can it remain efficient as ship detection precision declines?

This apparent conflict is clarified by analysing the competitive characteristics of multi-task learning. The shared encoder must serve both the detection head (focused on ship and IMO classification) and the reconstruction decoder (aimed at enhancing complex image features). The reconstruction goal and spatial limitations force the encoder to retain high-frequency attributes that are essential for distinguishing small IMO text, often at the expense of fine features that mainly aid in ship boundary identification.

Significantly, when ship detections are completely neglected ($N_{\mathrm{ship}}=0$), the spatial loss decreases to a constant (zero gradient), as elaborated in Section 3.4, and does not affect the training process. The detection loss continuously penalises overlooked ships and provides gradients to the shared encoder. Consequently, the decline in ship AP results from the transfer of features toward the minority IMO class instead of a deficiency in the spatial limitation. This compromise is acceptable in marine monitoring, as IMO numbers serve as the exclusive permanent ship identifier under SOLAS regulation XI-1/3. In contrast, overlooked ship detections can be corrected through temporal tracking or supplementary data sources such as AIS. The spatial loss continues to serve its intended function of removing physically inconsistent IMO detections. The relationship between ship detection accuracy and spatial loss effectiveness is further supported by the alternative spatial loss formulations evaluated in this study, with detailed results provided in Supplementary Table S11.

**Comparison with small-object and reconstruction-guided approaches:** Current small-object recognition techniques [24] emphasise multi-scale feature representation and improved feature extraction for small targets. However, these methodologies typically fail to directly consider the small textual characteristics of IMO numbers or the spatial relationship between an IMO number and its associated ship. Similarly, reconstruction-guided methodologies [63,64] show the importance of supplementary reconstruction goals for preserving identifying visual data; however, they are not specifically designed for marine IMO identification or ship–IMO geographical correlation. Our proposed architecture conceptually combines these complementary concepts with a domain-specific spatial constraint, simultaneously addressing class imbalance (IMOs represent just 1.2% of annotations) and spatial consistency. Taken together with the triangular training approach and skip-connected reconstruction decoder, this combination offers a complete solution specifically designed for the marine IMO detection challenge.

Furthermore, the proposed spatial regularisation incorporates a domain-specific geometric restriction that precisely defines the physical relationships among objects, requiring that IMO numbers must fall within a set of ship bounding boxes. Similar to data-driven solutions for managing imbalance, this restriction does not depend on class frequency but instead incorporates prior information into the learning process; as a result, the model is prevented from generating physically unreasonable predictions, including incorrect IMO detections outside maritime zones. Table 4 shows that using spatial loss improves IMO AP50-95 from 46.8% to 50.1% (+3.3 percentage points) and IMO precision from 93.6% to 97.8% (+4.2 percentage points), while recall remains the same at 91.7%. This approach improves localisation accuracy and better matches predicted bounding boxes to actual text areas. The enhancement is achieved without increasing inference costs, as the spatial limitation is applied only during training, with inference speed consistently maintained at 8.0 ms per image (Table 4).

A significant feature of the proposed approach is the compromise between IMO and ship detection efficiency. Although the model prioritises IMO accuracy, its ship detection declines. Table 4 shows that ship AP50-95 declines from 77.0% for YOLOv8s to 58.0% for the Hybrid (No Spatial) model, and subsequently to 55.6% for the full model, while ship recall falls from 92.6% to 79.6% and 77.8%, respectively. This trade-off makes sense in maritime monitoring, where IMO numbers function as unique ship identifiers and are accordingly more essential than identifying every ship incidence. In practical deployment, ship-level detection can often be improved with temporal tracking or multi-frame collection, while the absence of an IMO in a single frame leads to permanent information loss. Therefore, this shift in performance focus reflects task-aware optimisation rather than a limitation.

The legal and operational significance of the IMO number additionally supports the priority placed on IMO detection rather than ship-level recall. According to SOLAS regulation XI-1/3 [6], the IMO ship identification number is compulsory for all passenger ships exceeding 100 gross tonnage and all cargo ships exceeding 300 gross tonnage. The number must be permanently imprinted on the ship’s body or superstructure, included on all relevant ship certifications; importantly, it remains constant throughout the ship’s lifespan despite changes in name, ownership, or flag. This legal existence establishes the IMO number as the ship’s exclusive and dependable long-term identity.

In practical maritime surveillance, the implications of overlooking an IMO number differ significantly from those of failing to detect a ship. A failure to detect a ship can be rectified utilising temporal tracking across camera frames, radar information, or supplementary data sources such as the Automatic Identification System (AIS), which autonomously delivers ongoing position, identity, and tracking details for ships. AIS is required by SOLAS regulation V/19 [65] for all ships with 300 gross tonnage and above engaged in international travel, and for all passenger ships; however, if the IMO number is not recorded in a particular frame, especially while the ship is in range or at a crucial identification phase, this unique identification chance is permanently lost since the ship can drift out of view or change direction. Thus, port authorities and marine security personnel emphasise accurate identification of the unique IMO identifier rather than flawless ship recall, as the former confirms a ship’s legal identity while the latter mainly supports situational awareness. This practical situation based on the SOLAS-mandated identification standard justifies our design decision to emphasise IMO detection precision even if at the cost of compromising some ship-level detection capabilities.

In comparison to standard object detectors, covering both single-stage (YOLOv8s, RetinaNet, EfficientDet) and two-stage (Faster R-CNN) models as well as transformer-based methodologies (DETR), a clear trend is evident. As seen in Figure 8 and summarised in Table 4, our Hybrid+Spatial model achieves the highest IMO AP50-95 (50.1%) compared to all other approaches, beating RetinaNet (39.0%), Faster R-CNN (40.6%), DETR (36.7%), and EfficientDet (16.5%). All baseline models perform far better on ship detection than on IMO detection. This indicates that architectural enhancements are insufficient to correct the imbalance between substantial entities (ships) and tiny text-based objectives (IMO numbers). Specifically, techniques aimed at addressing class imbalance, such as focal loss in RetinaNet, do not fully reduce the problem, suggesting that the limitation stems from a lack of structural constraints and procedures for feature preservation. Similarly, EfficientDet’s low performance in IMO identification underscores the issue of multi-scale feature fusion alone being insufficient for precise text localisation.

The proposed framework ultimately delivers practical benefits beyond accuracy. The combined encoder and reduced detection process allow for fast inference, with the Hybrid+Spatial model achieving the most rapid inference time of 8.0 ms per image (Table 3), beating YOLOv8s (10.0 ms), RetinaNet (84.6 ms), Faster R-CNN (98.8 ms), DETR (69.9 ms), and EfficientDet (17.5 ms). This efficiency, along with enhanced robustness for small-object identification and physically consistent predictions, enables deployment in monitoring and observation systems. However, limitations remain. The small dataset includes only 297 images, which limits extensive analysis across different marine situations, ports, camera systems, and environmental circumstances. Although we implemented transfer learning, data augmentation, 5-fold cross-validation, and reconstruction-based regularisation to reduce overfitting, the dataset remains somewhat limited for training deep neural networks with additional reconstruction and regularisation branches. This may limit the model’s ability to adapt to novel locations, diverse camera viewpoints, or harsh climatic conditions that are not included in the training sample. Moreover, the spatial regularisation loss relies on the forecasted ship boxes; as mentioned in the Limitations and Failure Scenarios of Spatial Regularisation subsection, localisation inconsistencies may be transmitted through optimisation when ships overlap or are obscured. Assessment on other public maritime datasets may strengthen the generalisation of our conclusions. To confirm the robustness of the proposed framework in real marine surveillance systems, future investigations should examine large datasets that include various types of ports, camera systems, and environmental conditions.

**Practical impact and deployment considerations:** Although the suggested architecture showed strong efficiency on our selected dataset, certain elements need to be verified before practical implementation in real maritime surveillance systems. The initial assessment is limited to a single dataset of 297 images from the Baltic Shipping website, which may not fully represent the variety of ports, camera technologies, and environmental conditions found in maritime activities worldwide. Second, the model has not been evaluated under unfamiliar conditions, camera angles, or severe weather scenarios beyond the clear, foggy, and overcast situations represented in the training dataset. Third, the spatial regularisation loss requires ship boundary boxes, which may not be available in every situation, as elaborated in the Limitations and Failure Scenarios of Spatial Regularisation subsection. Consequently, although our findings are promising and demonstrate the capabilities of reconstruction-guided feature learning with spatial regularisation for maritime IMO detection, comprehensive large-scale validation across various ports, camera systems, and environmental conditions is necessary before implementation in operational systems. We highlight that this research serves as a proof-of-concept establishing a basis for subsequent efforts in real maritime surveillance applications.

**Failure case analysis:** Although overall performance is robust, the proposed approach reveals specific failure modes that offer valuable insights for future enhancements. In situations involving overlapping ships, the spatial loss may sometimes assign an IMO number to the wrong ship body when two ships are at close range. Instances of significant obstruction where deck equipment or structural components block IMO numbers may occasionally cause the detector to fails to identify the IMO number even when the spatial criteria are met. However, as confidence in ship detection decreases, the spatial loss may apply containment generated from an incorrect ship bounding box, resulting in less than ideal IMO localisation. These failure patterns underscore the dependence of the spatial loss on precise ship forecasts and the need for instance-level connection strategies in future attempts. The Limitations and Failure Scenarios of Spatial Regularisation subsection incorporates a comprehensive analysis of these limitations and potential enhancements.

### 6.2. Conclusions

This study has examined the challenges of marine object recognition caused by significant class imbalance and spatial inconsistency between ships and IMO numbers. To address these challenges, we introduce a hybrid autoencoder–YOLO system that combines reconstruction-based representation learning with spatial regularisation. The principal innovations of our methodology consist of: (1) reconstruction-driven feature learning, which pushes the common encoder to maintain sophisticated spatial features that are vital for identifying tiny IMO numbers; (2) differentiable spatial regularisation, which introduces the domain-specific requirement that IMO numbers must be located within ship bodies; and (3) a three-dimensional training framework that enhances optimisation stability by slowly combining reconstruction and spatial goals. This integration addresses class imbalance and geographical irregularity within a unified framework. The model combines a standard YOLOv8-based encoder with a skip-connected decoder for additional reconstruction, along with a spatial constraint that requires physically accurate associations between ships and IMO locations. The two-phase training technique includes autoencoder pretraining followed by sequential optimisation of detection and reconstruction with spatial supervision. The experimental outcomes support the two primary contributions put forward in this study:

#### Contribution 1: Hybrid Autoencoder—YOLO Architecture

The additional reconstruction branch enhances feature learning for the minority IMO class. Relative to the YOLOv8s baseline, Table 4 shows that the hybrid model without spatial loss enhances IMO AP50-95 from 42.3% to 46.8% (+4.5 percentage points), IMO recall from 87.5% to 91.7% (+4.2 percentage points), and IMO F1 from 89.4% to 92.6% (+3.2 percentage points). This confirms that reconstruction-based pretraining relying on the shared encoder can maintain the precise spatial information crucial for identifying small and low-contrast IMO text.

#### Contribution 2: Differentiable Spatial Regularisation

The spatial containment loss supports the requirement that IMO numbers must reside within ship bodies. Including this term enhances IMO AP50-95 from 46.8% to 50.1% (+3.3 percentage points) and raises IMO precision from 93.6% to 97.8% (+4.2 percentage points) while preserving recall at 91.7% (Table 4). This result shows that the spatial loss eliminates physically inconsistent detections and enhances bounding box alignment, thereby addressing the spatial inconsistency issue described in the Introduction. The comprehensive Hybrid+Spatial model achieves 50.1% IMO AP50-95, 97.8% IMO precision, and 94.6% IMO F1-score on the test set, a significant improvement compared to conventional detectors such as RetinaNet (39.0%), Faster R-CNN (40.6%), DETR (36.7%), and EfficientDet (16.5%) in the demanding IMO category (Figure 8). Although ship detection shows a trade-off (ship AP50-95 declines from 77.0% to 55.6%), this appears to be practically acceptable, as IMO numbers serve as the exclusive legally-required permanent identifier for ships under SOLAS regulation XI-1/3 [6], which makes their accurate detection more crucial than overall ship-level recall in maritime surveillance.

The proposed model achieves the fastest inference speed at 8.0 ms per image (Table 3), beating YOLOv8s (10.0 ms), RetinaNet (84.6 ms), Faster R-CNN (98.8 ms), DETR (69.9 ms), and EfficientDet (17.5 ms). This confirms that the hybrid architecture outperforms the others in accuracy and efficiency for real-time maritime applications. Ablation analyses on bottleneck design, spatial loss formulations, and training methods (see Supplementary Tables S8–S12) support our architectural and training choices, confirming their suitability for marine IMO detection. Statistical verification through five-fold cross-validation, with confidence intervals and paired hypothesis testing (p < 0.05), confirms that the noted performance enhancements are statistically significant and generalisable.

The proposed design shows significant promise in minimising the practical deployment difference caused by class imbalance and geographical inconsistency. However, we recognise that extensive validation across various ports, camera technologies, and environmental conditions is essential prior to implementation in operational maritime surveillance, port surveillance, and autonomous navigation systems. This research serves as a validation that establishes a basis for subsequent efforts in effective maritime IMO identification. To improve the relationship between ship and IMO performance and confirm the proposed framework’s flexibility across operational maritime circumstances, subsequent research will explore larger datasets including various ports, camera technologies, and environmental factors alongside video-based tracking and adaptive weighting techniques.

## Figures and Tables

**Table 1:** Comparison between existing approaches and the proposed framework across key dimensions.

Approach	Small Obj.	Scene-Text	Spatial	Maritime	Class Imbal.
General detectors (YOLOv8 [13], RetinaNet [43], Faster R-CNN [45])	Limited	No	No	Limited	Limited
Small object detection methods (SODFPN-YOLO [24], YOLOv8-sea [23], SeaLSOD-YOLO [22])	Yes	No	Limited	Limited	Limited
Scene-text detection methods (EAST [30], CRAFT [31], TextSnake [32])	No	Yes	No	Limited	No
Multi-task learning with autoencoders (AE-YOLO [39], Erkan et al. [40])	Limited	No	No	No	Limited
**Ours (Hybrid Autoencoder-YOLO + Spatial)**	**Yes**	**Yes**	**Yes**	**Yes**	**Yes**

**Table 2:** Average values of the autoencoder reconstruction metrics for ship datasets.

Metric	PSNR (dB)	SSIM	LPIPS
Autoencoder	26.18	0.8721	0.2377

**Table 3:** Inference speed comparison on NVIDIA A100 GPU (batch size = 1).

Model	Inference Time (ms/Image)
YOLOv8s	10.0
RetinaNet	84.6
Faster R-CNN	98.8
DETR	69.9
EfficientDet	17.5
**Hybrid+Spatial**	**8.0**

**Table 4:** Performance comparison of YOLOv8s, Hybrid (No Spatial), and Hybrid+Spatial. A ✓ indicates that the corresponding component is enabled.

Model	Auto.	Shared	Spatial	Class	AP50	AP50-95	Prec.	Rec.	F1	Time
						(%)	(%)	(%)	(%)	(ms)
YOLOv8s				IMO	85.3	42.3	91.3	87.5	89.4	10.0
				Ship	92.5	77.0	100.0	92.6	96.2	
	–	–	–	Overall	88.9	59.7	95.7	90.0	92.8	
Hybrid (No Spatial)				IMO	89.9	46.8	93.6	91.7	92.6	8.0
				Ship	72.2	58.0	100.0	79.6	88.7	
	✓	✓	✓	Overall	81.3	52.4	96.8	85.7	90.7	
**Hybrid+Spatial**				**IMO**	**90.7**	**50.1**	**97.8**	**91.7**	**94.6**	**8.0**
				**Ship**	**72.2**	**55.6**	**97.7**	**77.8**	**86.6**	
	✓	✓	✓	**Overall**	**81.7**	**52.9**	**97.8**	**84.8**	**90.6**	

Note: Overall metrics are macro averaged over both classes. Inference time is the average per image on an NVIDIA A100 GPU (batch size 1).

## Data Availability

The original contributions presented in this study are included in the article and Supplementary Materials. Further inquiries can be directed to the corresponding author.

## Supplementary Materials

The following supporting information can be downloaded at: https://www.mdpi.com/article/10.3390/jimaging12090433/s1, Table S1: Encoder architecture (EncoderWithSkips); Table S2: Decoder architecture (SkipDecoder); Table S3: Parameter breakdown of the full hybrid model; Table S4: Main training and evaluation hyperparameters; Table S5: Spatial loss weight schedule; Table S6: Confidence threshold schedule; Table S7: Sensitivity analysis of reconstruction weights; Table S8: Impact of bottleneck dimension; Table S9: Comparison of spatial loss formulations; Table S10: Comparison of training strategies; Table S11: Comparison of IMO and ship detection performance; Table S12: Cross-validation performance statistics; Table S13: Dataset composition statistics.

## Abbreviations

The following abbreviations are used in this manuscript:

IMO	International Maritime Organisation
YOLO	You Only Look Once
CNN	Convolutional Neural Network
AIS	Automatic Identification System
SOLAS	Safety of Life at Sea
AP	Average Precision
IoU	Intersection over Union
PSNR	Peak Signal-to-Noise Ratio
SSIM	Structural Similarity Index
LPIPS	Learned Perceptual Image Patch Similarity
Grad-CAM	Gradient-weighted Class Activation Mapping
