# Supplementary material for: A Hybrid Autoencoder YOLO Framework with Spatial Regularization for Rapid Small Maritime Object Detection
Source: J Imaging. 2026 Sep 10;12(9):433. doi: 10.3390/jimaging12090433 (PMC13608120; doi:10.3390/jimaging12090433)
Supplement: Supplementary file 1 [file jimaging-12-00433-s001.zip › jimaging-4467052-supplementary.pdf]

Journal of Imaging

MDPI

Article

A Hybrid Autoencoder YOLO Framework with Spatial Regularization for Rapid Small Maritime Object Detection

M Mohidul Hossain Khan [1], Qiwei Hu [2], Radhakrishna Prabhu [1], Haiyong Zheng [3], Huagui Huang [2] and Zonghua Liu [1,*]

[1] School of Computing, Engineering and Technology, Robert Gordon University, Aberdeen AB10 7QB, UK
[2] National Engineering Research Center for Equipment and Technology of Cold Strip Rolling, Yanshan University, Qinhuangdao 066004, China
[3] College of Electronic Engineering, Ocean University of China, Qingdao 266003, China
* Correspondence: z.liu3@rgu.ac.uk

S1. Network Architecture Details

The autoencoder has a shared weight architecture: the encoder includes the initial nine layers of YOLOv8s (indices 0–8), wrapped within EncoderWithSkips; the decoder is a customised U-Net style SkipDecoder. In the hybrid and spatial models, the encoder layers share weights with the YOLO detection backbone, allowing parallel feature learning. The input and reconstruction resolutions are 640 × 640 × 3.

S1.1 Encoder (EncoderWithSkips)

The encoder wraps YOLOv8s backbone layers 0–8 (the SPPF layer at index 9 is excluded to match the autoencoder design) (Table S1). At layers 2 and 4, intermediate feature maps are saved as skip connections for the decoder.

Table S1. Encoder architecture (EncoderWithSkips).

Layer	Module	Output Shape	Stride	Notes
Input	—	640×640×3	1	RGB image
0	Conv 3×3, s=2	320×320×32	2	—
1	Conv 3×3, s=2	160×160×64	4	—
2	C2f	160×160×64	4	Skip 1 → Decoder
3	Conv 3×3, s=2	80×80×128	8	—
4	C2f	80×80×128	8	Skip 2 → Decoder
5	Conv 3×3, s=2	40×40×256	16	—
6	C2f	40×40×256	16	—
7	Conv 3×3, s=2	20×20×512	32	—
8	C2f	20×20×512	32	Bottleneck → Decoder

Encoder parameters: 4,422,816 (∼4.42M)

S1.2 Decoder (SkipDecoder)

The decoder follows a U-Net style architecture with two skip connections from encoder layers 2 and 4. All convolutions use 3×3 kernels (except 1×1 projection layers), Batch Normalisation, and SiLU activation. The final layer uses Sigmoid to constrain pixel values to [0,1].

Decoder parameters: 2,262,182 (∼2.26M)

Full Autoencoder (Encoder + Decoder) parameters: 6,684,998 (∼6.68M)

Check for updates

Academic Editor: Qingyong Li

Received: 14 July 2026
Revised: 28 August 2026
Accepted: 30 August 2026
Published: 10 September 2026

Copyright: © 2026 by the authors. Licensee MDPI, Basel, Switzerland. This article is an open access article distributed under the terms and conditions of the Creative Commons Attribution (CC BY) license.

Table S2. Decoder architecture (SkipDecoder).

Block	Operation	Channels	Size	Notes
Bottleneck	3× Conv-BN-SiLU	512→256→128→64	20×20	Reduces to 64 ch
Up1	Upsample ×2 + Conv	64→128	40×40	—
Skip fusion 4	Concat+Conv (skip from L4)	128+128→256→128	80×80	Skip from L4
Up2	Upsample ×2 + Conv	128→128	80×80	Upsample to 80×80
Skip fusion 2	Concat+Conv (skip from L2)	128+64→192→64	160×160	Skip from L2
Up3	Upsample ×2 + Conv	64→64	320×320	Upsample to 160×160
Up4	Upsample ×2 + Conv	64→32	640×640	Final upsampling
Final	Conv-BN-Sigmoid	32→3	640×640	Output reconstruction

S1.3 Integration with YOLO (Hybrid / Spatial Model)

In the hybrid framework, the encoder layers (0–8) are shared between the detection backbone and the reconstruction branch. The YOLO detection head and the decoder operate in parallel on the shared features.

Table S3. Parameter breakdown of the full hybrid model.

Component	Description	Parameters
YOLOv8s (2-class)	Full detector (backbone + neck + head)	11,136,374 (∼11.14M)
SkipDecoder	Reconstruction branch (added)	2,262,182 (∼2.26M)
**Total (unique, shared backbone)**	Encoder layers 0–8 shared; decoder added	∼**13.40M**

The shared encoder does not add extra parameters beyond the YOLO backbone. Only the decoder adds approximately 2.26M additional parameters, representing a ∼20% increase over the base YOLOv8s model (11.14M → 13.40M).

S1.4 Pretraining

The encoder and decoder are initialised from a pretrained autoencoder checkpoint (best_model.pth), trained on the maritime ship dataset using the combined reconstruction loss (L1 + edge-aware + multi-scale, as described in Section 3.3 of the main manuscript).

Weight mapping:

- Encoder weights are mapped into YOLO backbone layers 0–8 (the shared encoder).
- Decoder weights are loaded directly into SkipDecoder.

Training details for pretraining:

- Optimiser: Adam, learning rate 1×10^{-4}, weight decay 1×10^{-5}.
- Epochs: 300 (early stopping based on validation loss).
- Data: Mixed augmentation (DAE + YOLO-style, 50/50 mix) with clean letterbox targets.
- Loss: Combined reconstruction loss (L1 + edge + multi-scale).

This pretrained checkpoint provides a robust initialisation for the hybrid model, enabling faster convergence and improved feature learning for the minority IMO class.

S2. Hyperparameter Settings

All values listed relate to the proposed Hybrid + Spatial model. The Hybrid baseline uses identical settings, with the exception that the spatial loss is deactivated ($\lambda = 0$, removing the spatial term).

S2.1 Main Training and Evaluation Hyperparameters

Table S4. Main training and evaluation hyperparameters.

Hyperparameter	Setting	Notes
Optimizer	Adam	Phase 1: YOLO head only; Phase 2+: 3 groups
Learning rate (YOLO head)	1×10^{-3}	Epochs 1–200 (neck + head, layers 9+)
Learning rate (encoder/backbone)	1×10^{-5}	Epochs 21–200 (layers 0–8; frozen Phase 1)
Learning rate (decoder)	1×10^{-5}	Epochs 21–200 (frozen Phase 1)
LR scheduler	Cosine annealing	$T_{\max} = 200$, $\eta_{\min} = 1 \times 10^{-7}$
Batch size	8	Limited by GPU memory
Weight decay	0.0005	Same as YOLOv8 default
Number of epochs	500	5-fold CV; best by validation loss
Input image size	640×640	Letterbox preprocessing
Detection loss weight (α)	1.0	DETECTION_WEIGHT
Reconstruction loss weight (β)	1.0	RECONSTRUCTION_WEIGHT (0 in Phase 1)
Spatial loss weight (λ)	Scheduled	See Table S5; adaptive halving if $>$ 0.5
Confidence threshold (τ) – spatial	Scheduled	See Table S6 (training only)
Confidence threshold – inference	0.50	Input filtering
IoU threshold	0.45	Standard YOLOv8 default; per-class NMS

S2.2 Spatial Loss Weight Schedule (λ)

The spatial weight λ is increased gradually to ensure the spatial constraint acts as an auxiliary regulariser in early training and becomes more influential later.

Table S5. Spatial loss weight schedule (λ).

Epoch Range	λ
0–9	0.01
10–19	0.02
20–29	0.05
30–39	0.10
40–49	0.15
50–59	0.20
60–69	0.25
70–79	0.30
80–89	0.35
90–99	0.40
100–199	0.45
200	0.50

Adaptive rule: If $L_{\text{spatial}}/L_{\text{detect}} > 0.5$, λ is multiplied by 0.5 for that batch to prevent the spatial term from dominating.

S2.3 Total Loss Formulation (Phase 2+, Spatial Enabled)

The total loss for the spatial-aware hybrid training phase is defined as:

$$L_{\text{total}} = \alpha \cdot L_{\text{detect}} + \beta \cdot L_{\text{recon}} + \lambda \cdot L_{\text{spatial}} \quad \text{(S1)}$$

where:

- $\alpha = 1.0$ (detection loss weight),
- $\beta = 1.0$ (reconstruction loss weight),

- λ follows the schedule in Table S5.

S2.4 Confidence Threshold (τ) for Spatial Loss (Training)

The confidence threshold τ determines which predicted boxes are used in the spatial loss computation. A lower threshold early in training ensures the spatial loss receives gradients even when detection confidences are weak.

Table S6. Confidence threshold (τ) schedule for spatial loss (training only).

Epoch Range	τ	Purpose
0–19	0.02	Include weak early predictions so spatial term is non-zero
20–59	0.05	Default spatial box extraction
60–200	0.25	Stricter boxes; reduces noisy spatial gradients

Note: τ applies only when extracting predicted IMO and ship boxes for the spatial containment loss. It does not affect the final mAP evaluation, which uses a fixed confidence threshold of 0.50.

S2.5 Sensitivity Analysis of Reconstruction Loss Weights

To justify the chosen reconstruction loss parameters, we performed a sensitivity analysis varying λ_{edge} and w on Fold 4 of the validation split over 500 epochs.

Table S7. Sensitivity analysis of reconstruction weights (λ_{edge} and w) on IMO AP50-95.

Configuration	λ_{edge}	w	Val IMO AP50-95 (%)	Test IMO AP50-95 (%)
Reference (Ours)	0.30	0.25	45.67	44.85
Low Edge	0.10	0.25	43.85	44.85
High Edge	0.50	0.25	40.55	43.62
Low Multi-Scale	0.30	0.10	46.67	45.35
High Multi-Scale	0.30	0.50	44.62	44.46

The sensitivity analysis demonstrates the effect of varying the edge and multi-scale regularisation weights on IMO detection performance. Increasing the edge weight from 0.30 to 0.50 decreases test IMO AP50-95 from 44.85% to 43.62%, indicating that excessive emphasis on edge preservation can adversely affect detection of small IMO markings. Reducing the edge weight to 0.10 results in 44.85% test IMO AP50-95, showing that a moderate edge contribution provides a balanced configuration. For the multi-scale term, reducing the weight from 0.25 to 0.10 produces a slightly higher test IMO AP50-95 of 45.35%, whereas increasing it to 0.50 results in 44.46%. These results suggest that excessive weighting of either auxiliary term does not consistently improve IMO detection. The selected configuration ($\lambda_{\text{edge}} = 0.30$ and $w = 0.25$) therefore provides a balanced contribution from pixel-level, edge, and multi-scale reconstruction objectives without allowing the auxiliary terms to dominate the detection objective.

S3. Ablation of Architectural and Training Design Choices

To justify the architectural and training design choices, we performed three ablation studies on one validation fold (Folder 4) and the reserved test set (47 images).

S3.1 Bottleneck Dimension Analysis

We evaluated bottleneck parameters to analyse the balance between feature compression and information preservation for small-object detection. The encoder generates a compressed feature map with dimensions $C \times H \times W$. We evaluated configurations

of 128 × 20 × 20, 512 × 20 × 20 (our variant), and 512 × 40 × 40, maintaining all other architectural factors constant.

Table S8. Impact of bottleneck dimension on IMO detection performance (Folder 4, 500 epochs).

Bottleneck Dimension	Val IMO AP50-95 (%)	Test IMO AP50-95 (%)
128 × 20 × 20	45.90	46.86
512 × 20 × 20 (Ours)	45.67	44.85
512 × 40 × 40	40.69	44.27

The results show that while the smaller bottleneck (128 × 20 × 20) achieved a marginally better test IMO AP50-95 (46.86%) than the chosen 512 × 20 × 20 configuration (44.85%), the 512 × 20 × 20 bottleneck offers a better balance between feature compression and information preservation. The higher channel bottleneck preserves complex feature representations while avoiding the excessive compression of the 128-channel setup. Given the marginal performance difference, we keep 512 × 20 × 20 as the balanced configuration for the proposed framework.

S3.2 Comparison of Spatial Loss Formulations

We reviewed the suggested IoU-based confinement loss against two other geometric constraints: center-point containment and differentiable clipping. All models underwent training with an identical configuration on Folder 4 for a duration of 500 epochs.

Table S9. Comparison of spatial loss formulations on IMO detection (Folder 4, 500 epochs).

Spatial Loss Type	Val IMO AP50-95 (%)	Test IMO AP50-95 (%)
Center-point containment	39.38	38.56
Differentiable clipping	42.79	42.38
**IoU-based containment (Ours)**	**45.67**	**44.85**

The IoU-based containment loss achieves superior IMO AP50-95 across both validation and test datasets. Center-point containment results in decreased performance as it ignores partial overlap and spatial dimensions, simply verifying if the IMO center is situated within a ship's bounding box. Differentiable clipping achieves similar yet less satisfactory results while adding further hyperparameters. We consequently maintain IoU-based containment due to its straightforwardness and exceptional efficiency.

S3.3 Training Strategy Comparison

We evaluated the suggested three-phase training methodology (autoencoder pretraining → hybrid detection-reconstruction → spatial-aware training) in contrast to a singular stage end-to-end method where all losses are adjusted from the first epoch.

Table S10. Comparison of training strategies on IMO detection (Folder 4, 500 epochs).

Training Strategy	Val IMO AP50-95 (%)	Test IMO AP50-95 (%)
Single-Stage End-to-End	41.29	44.53
**3-Stage (Ours)**	**45.67**	**44.85**

The three-phase procedure achieves superior IMO AP50-95 relative to the single-phase method. The one-stage training experiences inconsistency during first epochs, as the spatial and reconstruction losses disturb the detection head prior to the formation of significant features. The pretraining phase offers a reliable starting point, while the gradual implementation of the spatial loss ensures that spatial constraints do not overshadow initial optimisation. Consequently, we maintain the three-stage approach.

S3.4 Comparison of IMO and Ship Detection Performance Across Spatial Loss Formulations

The relationship between ship identification precision and the performance of spatial loss is additionally supported by the other spatial loss models assessed in Section S3.2. Table S11 compares ship AP50-95 with IMO AP50-95 under different spatial limitations.

Table S11. Comparison of IMO and ship detection performance across spatial loss formulations (Test set).

Spatial Loss Type	IMO AP50-95 (%)	Ship AP50-95 (%)	IMO Precision (%)
Center-point containment	38.56	58.43	91.49
Differentiable clipping	42.38	59.27	100.00
**IoU-based containment (Ours)**	**44.85**	**55.60**	**97.80**

The IoU-based containment loss achieves a maximum IMO AP50-95 (44.85%) and IMO precision (97.8%), however provides the lowest ship AP50-95 (55.6%). In contrast, center-point containment maintains a superior ship AP (58.43%) but results in significantly lower IMO AP50-95 (38.56%). This opposite correlation confirms that the decline in ship AP is a direct result of increased spatial limitations allocating encoder resources toward IMO-related features.

S3.5 Statistical Validation

To evaluate the statistical dependability and applicability of the proposed framework, we examined the IMO AP50-95 performance over the five cross-validation folds. Table S12 includes the findings for the baseline YOLOv8s alongside the suggested Hybrid+Spatial model.

Table S12. Cross-validation performance statistics for IMO AP50-95 (5 folds).

Fold	YOLOv8s (%)	Hybrid+Spatial (%)
1	42.0	50.1
2	41.9	49.8
3	37.4	46.2
4	42.5	50.1
5	36.7	45.3
**Mean**	**40.1**	**48.3**
**Std. Dev.**	**2.7**	**2.4**
**95% CI**	**[37.7, 42.5]**	**[46.2, 50.4]**

The suggested Hybrid+Spatial model achieved a mean IMO AP50-95 of 48.3% across five folds, in contrast to 40.1% for the baseline YOLOv8s, indicating an enhancement of 8.2 percentage points.

To determine if the noted enhancement is statistically significant, we conducted a paired t-test contrasting the suggested model with the baseline over the five folds. The findings indicate a statistically significant enhancement (t-statistic $= 37.0$, p-value $= 3.17 \times 10^{-6}$), indicating that the performance improvements of the suggested framework are not related to random fluctuations. The non-overlapping confidence intervals further support this conclusion.

These statistical evaluations provide convincing evidence that the suggested reconstruction-guided feature learning and spatial regularisation methods produce substantial and transferable enhancements in maritime IMO detection.

S4. Data Collection and Dataset Construction

All images used in this study originate from Baltic Shipping. The collection encompasses a diverse range of real-world maritime conditions, including various lighting scenarios (low-light, overcast, sunny, and night), weather conditions (clear, foggy, and cloudy),

viewing angles (front, side, and oblique), and distances (close-up, medium-range, and far-away). Due to factors such as hull colour, surface damage, rust, occlusion by equipment or structural elements, and varying image resolution, IMO markings exhibit significant diversity in size, font style, positioning, contrast, and visibility. The dataset includes a wide variety of vessel types (cargo, tanker, fishing, and container ships) with IMO numbers ranging from very small and low-contrast to clearly visible, ensuring comprehensive coverage.

The original full-ship images have a width of approximately 660–1240 pixels (the WebP sources are 800 pixels wide). An initial pool of roughly 3,000 candidate ship images was reviewed through a two-stage screening process:

- In Stage 1, images were retained if the ship occupied more than 70% of the frame area, excluding those where the ship appeared too small, was captured from a distant angle, or was dominated by dock or shipyard infrastructure. This stage was carried out by Qiwei Hu, Hanxiao Li, Shuhao Lyu, and Hao Fan, and resulted in approximately 600 images.
- In Stage 2, Qiwei Hu, Hanxiao Li, and Shuhao Lyu manually curated the set, selecting only images with a clearly legible IMO number region. Images where the IMO could not be read clearly were excluded, yielding the final 297 annotated images used in this paper; all excluded images are retained on record.

Bounding boxes for both ships and IMO regions were drawn by M. Mohidul Khan using Roboflow, following a written annotation protocol, and were subsequently reviewed by M. Mohidul Khan and Zonghua Liu over 100% of the samples to ensure quality and consistency. As described in Section 4.1 of the main manuscript, 47 images are held out as a fixed test set prior to cross-validation, while the remaining 250 images are used for 5-fold cross-validation, with 200 images for training and 50 for validation per fold. All images were letterboxed to 640×640 to ensure uniform input dimensions.

Several limitations should be explicitly acknowledged:

- First, every retained image contains a visible and annotatable IMO number; frames with no visible IMO in real-world monitoring were excluded. Notably, approximately 608 ship images with no visible IMO were not used, meaning precision and recall metrics do not apply to that scenario.
- Second, Stage 2 excluded images where the IMO could not be read clearly due to occlusion, glare, motion blur, or other factors, resulting in a cleaner dataset than typical real-world port footage. Performance on these excluded cases is not evaluated in this study and is deferred to future work.
- Third, the screening process is inherently subjective: although the $> 70\%$ ship-occupancy rule provides a quantitative threshold, it was estimated visually by the annotators, and IMO readability is likewise a human judgment. Both stages rely on the annotators' experience, which may introduce some variability.

Table S13 summarizes the dataset composition across lighting conditions, weather conditions, and ship types.

https://doi.org/10.3390/jimaging12190433

Table S13. Dataset composition statistics (297 images).

Category	Subcategory	Count (Images)	Percentage (%)
**Lighting Condition**	Day / Clear	180	60%
	Night / Low-light	20	7%
	Overcast / Cloudy	60	20%
	Sunny / Bright	37	12%
	**Total**	**297**	**100%**
**Weather Condition**	Clear	180	60%
	Foggy / Misty	50	17%
	Cloudy	67	23%
	**Total**	**297**	**100%**
**Ship Type**	Cargo / Container	100	34%
	Tanker / Oil Tankers	80	27%
	Mid-Range Ships	60	20%
	Fishing Boats	40	13%
	Other	17	6%
	**Total**	**297**	**100%**

The collection includes images that were taken in various circumstances ensuring academic strength: around 60% during daylight/clear weather, 7% at night/low light, and 20% under overcast/cloudy conditions. Weather conditions include clear (60%), foggy (17%), and overcast (23%). The categories of ships includes cargo/container ships (34%), tankers (27%), mid-sized ships (20%), fishing ships (13%), and other boats (6%). This variety allows the model to predict across the diverse visual circumstances faced in actual marine monitoring. All bounding box annotations were executed by one principal annotator utilising Roboflow [51]. Every label underwent careful examination and validation to guarantee quality. Formal inter-annotator agreement metrics are unnecessary because of the single-annotator framework, yet quality assurance was confirmed through careful examination.

https://doi.org/10.3390/jimaging12190433
